# Sugarcane streak mosaic virus P1 protein inhibits unfolded protein response through direct suppression of *bZIP60U* splicing

**DOI:** 10.1371/journal.ppat.1011738

**Published:** 2023-10-26

**Authors:** Kun Zhang, Tianxiao Gu, Xiaowei Xu, Haifeng Gan, Lang Qin, Chenwei Feng, Zhen He

**Affiliations:** 1 Department of Plant Pathology, College of Plant protection, Yangzhou University, Yangzhou, Jiangsu Province, P. R. China; 2 Joint International Research Laboratory of Agriculture and Agri-Product Safety of Ministry of Education of China, Yangzhou University, Yangzhou, P. R. China; 3 Jiangsu Key Laboratory for Microbes and Functional Genomics, Jiangsu Engineering and Technology Research Center for Microbiology, College of Life Sciences, Nanjing Normal University, Nanjing, P. R. China; Agriculture and Agri-Food Canada, CANADA

## Abstract

The unfolded protein response (UPR) is a cell-designated strategy that maintains the balance of protein folding in the endoplasmic reticulum (ER). UPR features a network of signal transduction pathways that reprogram the transcription, mRNA translation, and protein post-translational modification to relieve the ER stresses from unfolded/misfolded proteins. Infection with plant viruses can induce the UPR, and activated UPR often promotes plant viral infections in turn. However, the mechanism used by plant viruses to balance UPR and achieve robust infection remain largely unknown. In this study, P1^SCSMV^ was identified as a virus-encoded RNA silencing suppressor (VSR). Heterologous overexpression of P1^SCSMV^ via potato virus X (PVX) was found lead to programmed cell death (PCD) in *Nicotiana benthamiana*. Furthermore, P1^SCSMV^ was also found to inhibit the PVX infection-triggered UPR by downregulating UPR-related genes and directly induced the distortion and collapse of the ER polygonal meshes on PVX-P1^SCSMV^ infected *N*. *benthamiana*. Moreover, self-interaction, VSR activity, UPR inhibition, and cell death phenotype of P1^SCSMV^ were also found to be dependent on its bipartite nuclear localization signal (NLS) (^251^RKRKLFPRIPLK^262^). P1^SCSMV^ was found to directly bind to the stem-loop region of *NbbZIP60U* via its NLS and inhibit the UPR pathways, ultimately resulting in a PCD phenotype in PVX-P1^SCSMV^ infected *N*. *benthamiana* leaves. This study also revealed the balancing role of potyviruses encoded P1^SCSMV^ in the UPR pathway to achieve robust viral infection. This may represent a novel virulence strategy for plant viruses.

## Author summary

Unfolded protein response (UPR) is a cell designated strategy that maintains the balance of protein folding in the endoplasmic reticulum (ER). UPR contains a network of signal transduction pathways that reprogram the transcription, mRNA translation, and protein post-translational modification to relieve the ER stresses from unfolded/misfolded proteins. Plant viruses’ infection induce the UPR, and activated UPR often promotes plant viral infection in turn. However, it is unclear how plant viruses balance the UPR to achieve robust infection. In this study, P1^SCSMV^ was identified as a virus-encoded RNA silencing suppressor (VSR). P1^SCSMV^ was found to inhibit PVX infection-triggered UPR by directly binding to the stem-loop region of *NbbZIP60U* with its bipartite nuclear localization signal (NLS) and inhibits the UPR pathways, thus leading to the distortion and collapse of the ER polygonal meshes and the cell-death phenotype on PVX-P1^SCSMV^ infected *N*. *benthamiana*. The self-interaction, VSR activity, UPR inhibition, and cell-death phenotype of P1^SCSMV^ are dependent on its NLS (^251^RKRKLFPRIPLK^262^). This study reveals the balancing role to the UPR pathway by potyviruses encoded P1^SCSMV^, which enriched the multi-functionality of the viral-encoded VSRs. This may represent a novel virulence strategy by plant viruses.

## Introduction

RNA silencing is used to control the antiviral immune response in a diverse range of eukaryotes. During viral infection, the host dicer type III nuclease (RNase III) processes the viral replication intermediate dsRNA into small interfering RNAs (siRNAs). These siRNAs are then loaded onto the Argonaute protein (AGO) to guide specific viral clearance by RNA silencing [1]. However, most plant viruses encode RNA silencing suppressor (VSRs) that inhibit different branches of the host antiviral RNA silencing pathways, and thereby achieve robust multiplication [1–3]. Previous studies have shown that most VSRs can interact with themselves and form dimers/polymers. This enables binding to siRNA/dsRNAs to block host antiviral RNA silencing response *in vivo*. For instance, barley stripe mosaic virus γb protein interacts with itself via the C-terminal coil-coiled domain, which is required for siRNA binding and inhibition of the RNA induced silencing complex (RISC) formation [4,5]. Likewise, cucumber mosaic virus 2b protein interacts with itself. Polymers of 2b are essential for dsRNA binding and inhibition of the RNaseIII-like dicer’s dicing of viral replication intermediate dsRNA and RISC formation by binding to Ago1 [6]. Hc-Pro of *Potyvirus* is also a suppressor of antiviral RNA silencing and forms homodimers *in vivo*, inhibiting dicer processing by dsRNA binding [7,8]. The multifunctional P19 of the tomato bushy stunt virus also interacts with itself to predominantly form dimers via the central region, which sequesters small RNA duplexes and prevents RISC assembly [9]. The NS3 protein of *Tenuivirus* also undergoes self-interaction. This is required for small RNA duplexes binding and suppression of antiviral RNA silencing [10,11]. The βC1 encoded by tomato yellow leaf curl China beta-satellite is capable of forming multimeric complexes *in vitro* and *in vivo* to upregulate the endogenous RNA-silencing suppressor rgs-CaM that inhibits the expression of RNA dependent RNA polymerases 6 (RDR6), and lead to SGS3 degradation [12,13]. However, while P1^SCSMV^ may act as an RNA silencing suppressor, questions remain about its specific mechanism. It is unknown whether P1^SCSMV^ self-interaction/polymerization is required to suppress host antiviral RNA silencing. Further exploration is needed to understand how P1^SCSMV^ actually works *in vivo*.

The unfolded protein response (UPR) is a highly conserved pathway that helps manage ER stress imposed from secretory demands placed on the ER environmental factors [14]. The UPR orchestrates adaptation to ER stress, rescues healthy ER function, and prolongs cell viability [15]. The molecular mechanisms of UPR have been extensively studied in yeast and animals [16]. In yeast, the UPR is controlled by the type I transmembrane ER protein (IRE1) signaling pathway. In plant, two UPR pathways were identified so far, the IRE1-bZIP60-associated pathway and the bZIP28/bZIP17-associated pathway [17]. In the IRE1-bZIP60-associated pathway, IRE1 is an ER-resident transmembrane protein that possesses kinase activity and senses ER stress through its luminal domain [18]. In response to ER stress, IRE1 becomes activated directly or indirectly through interactions with unfolded proteins. This activation leads to IRE1 auto-phosphorylation and self-interaction. IRE1 then splices *bZIP60U* mRNA [16]. IRE1-mediated splicing generates *bZIP60S*. Translated bZIP60S acts as a transcription factor by entering the cell nucleus and regulating the expression of downstream target genes associated with protein folding and alleviation of ER-stress [19]. A properly functioning UPR mediated by the IRE1-bZIP60 pathway enhances cell survival under stressful conditions by optimizing protein folding process in the ER secretory pathway and preventing programmed cell death from occurring when stress becomes severe [19]. Plant viruses are obligate intracellular parasites that rely on host factors in their life cycle [20]. Robust viral activities enhance protein synthesis via ribosomes located in the ER, and the overwhelming loading of unfolded/misfolded proteins is finally recognised by the ER-resident IRE1 or bZIP17/bZIP28[19]. Several classical models have been proposed for the recognition and activation of ER sensors or UPR pathways. [21]. Recognition between the receptor and ligand must be chemically, specific, and physically precise. Otherwise the UPR system must be disordered [16].

Several studies have shown that plant viral infections can activate the UPR, and the activated UPR can promote viral replication and infection. For instance, the 6K2 of potato virus Y (PVY) and turnip mosaic virus (TuMV) [17,22], triple gene block 2 and 3 (TGB2 and TGB3) of potato virus X (PVX) [22–24], P10 of rice-black-streak dwarf virus (RBSDV) [25], P11 of the garlic virus X (GarVX) [15], the TGB3 of plantago asiatica mosaic virus (PlAMV) [26], and the βC1 of *Geminivirus* were able to anchor or localize to the ER and activate the UPR. Here, UPR activation plays pro-viral roles in the infection of these viruses by transcriptional induction of ER chaperone and protein-folding genes, such as *BiP*, *CRT*, and *PDI* [27]. Although extensive studies have been performed to explore the interactions between plant viruses and the host UPR, the interplay between a single viral protein and UPR components remains largely unknown.

Sugarcane streak mosaic virus (SCSMV) is a member of the *Poacevirus*, genus within the family *Potyviridae* [28]. It is an emerging cause of sugarcane mosaic disease worldwide [29,30], resulting in significant effects on sugar and ethanol production in sugarcane cultivation regions [31]. SCSMV has a long single-stranded, positive sense RNA genome of approximately 10,000 nucleotides (nt) [32]. The genomic RNA is encapsidated in a flexuous filamentous particle (890 X 15 nm), and encodes a large polyprotein that is further processed to form ten mature proteins by hydrolytic cleavage (P1, HC-Pro, P3, 6K1, CI, 6K2, VPg, NIa-Pro, NIb, and Coat Protein) [33,34]. P1^SCSMV^ has been shown to be the most distinct protein among *Potyviridae* members, representing evolutionary variation contributing to the viral adaptation to a wide range of host species [35]. The P1^SCSMV^ of *Poacevirus* has also been shown to be a classical VSR, [36,37], plays a disease-enhancing role in conditions of *cis*-heterogeneous-mediated expression by potato virus X (PVX) in *Nicotiana benthamiana* [38,39]. The P1^SCSMV^ protein of the triticum mosaic virus, which is the type virus of the genus *Poacevirus*, can inhibit host antiviral RNA silencing by binding to the double-stranded RNA (dsRNA) [40]. Hence, P1^SCSMV^ of SCSMV clearly plays an essential role in the plant immune response and physiological state alterations during multidimensional virus-plant interactions. However, the underlying mechanisms are still unknown, and exploring the characteristics of P1^SCSMV^ is important to develop better control strategy in the sugarcane production field.

Here, P1^SCSMV^ was confirmed to function as a classical VSR that suppresses both local and systemic antiviral RNA silencing. We heterologously overexpressed P1^SCSMV^ via recombinant PVX, and found that P1^SCSMV^ enhanced viral symptoms, increased recombinant virus accumulation, and promoted PCD. Furthermore, P1^SCSMV^ was also found to inhibit PVX-GFP infection-triggered UPR by downregulating UPR-related genes. Transient overexpression analyses further showed that P1^SCSMV^ suppresses the expression of the UPR marker genes *CAM* and *BLP4*. We also found that P1^SCSMV^ localized to both the nucleus and cytoplasm, and that the bipartite nuclear localization signal (NLS) (^251^RKRKLFPRIPLK^262^) was required for self-interaction, VSR activity, UPR inhibition, and the PCD phenotype. Furthermore, compared with PVX-GFP, silencing of the nuclear translocation machinery (*NbImp*. *α* and *NbImp*. *β*) decreased the cell-death intensity in PVX-P1^SCSMV^ infection. On the other hand, silencing of the UPR marker genes *NbZIP60* and *NbBLP4* greatly promoted cell-death. We also found that P1^SCSMV^ could directly induce distortion and collapse of ER polygonal meshes. Moreover, *in vivo* RNA-immunoprecipitation assays and *in vitro* electrophoretic mobility shift assays (EMSAs) also demonstrated binding of P1^SCSMV^ to the splicing region of *NbbZIP60U* through its NLS peptide, and inhibited IRE1-associated UPR signaling pathways, to results in cell-death in PVX-P1^SCSMV^ infection. These findings provide a novel perspective on how the viral single protein (P1^SCSMV^) inhibits IRE1-associated UPR signaling pathways to display viral pathogenicity. Our results highlight the multifunctionality of virus-encoded VSRs and may guide further studies on the potyvirus resistance and high-yield sugarcane cultivation.

## Method and materials

### Vector construction

PVX-GFP and PVX-P1^SCSMV^ were constructed as previously described [39]. Briefly, the original pGreen208 vector expressed the infectious cDNA of PVX-GFP, and the open reading frame of P1^SCSMV^ was cloned into the pND108 vector [41]. The primers used are listed in **S1 Table.**

### Plant growth conditions and virus inoculation

A climate-controlled chamber with a light/dark photoperiod of 16/8 h, and an 80 mmol/m^2^ light intensity at 24°C was used to cultivate *N. benthamiana* as described previously [39]. The recombinant viruses PVX-GFP and PVX-P1^SCSMV^ were inoculated into *N*. *benthamiana* via *Agrobacteria*-mediated infiltration of the leaves. The procedures are detailed below.

### Agroinfiltration and GFP imaging to investigate the RNA silencing suppressor activity

*Agrobacterium* containing the binary vectors were cultured in Luria-Bertani (LB) liquid medium with 100 mg/L Kan and 25 mg/L Rif. Cells were harvested by centrifugation at 3000 X g for 10 min after culture at 220 rpm shaking and 28°C for 12 h. *Agrobacterium* suspension buffer was used to suspend the cells as previously described [5]. The OD_600_ of the cells was adjusted to 0.5, and then the suspensions were incubated at 28°C for at least 2 h before infiltration.

For VSR activity evaluation, *Agrobacterium* containing the 35S-driven single-strand GFP (ssGFP) cassette combined with equal amounts of other suspensions harboring the P1^SCSMV^ cassette and its different derivatives, with an optical density (OD_600_) of 0.5, was co-infiltrated into the abaxial leaves of *N*. *benthamiana*. After 3-day-post-infiltration (dpi) and at 5 dpi, the green fluorescence was recorded under a longwave ultraviolet lamp (B-100AP/R, UVP) by a digital camera (EOS 80D, Canon) with a yellow filter (Gelatin filter No. 15, Kodak). The primers used are listed in **S1 Table.**

### Total RNA extraction and reverse-transcription PCR detection

Total RNA was extracted from the infiltrated-leaves using TRIzol Reagent (Cat. No., 15596018, Thermo Fisher Scientific, Shanghai, China). Recombinant DNase I (RNase-free) kit (Code No, 2270A, Takara, Dalian, China) was used to digest genomic contaminations in total RNAs. Then, the cDNA was obtained by reverse transcription reaction using the M-MLV reverse transcriptase (Sigma-Aldrich, Shanghai, China) from the 1.0 μg of total RNA as described [39]. The primers used are listed in **S1 Table.**

### Western blot assay and Coomassie brilliant blue (CBB) staining

Total tissue proteins were extracted using equal volumes of protein loading buffer as described previously [5]. Then, the samples were incubated in boiling water for 10 min and centrifuged at 12,000 X g for 10 min. The supernatant was loaded onto a 12.5% sodium dodecyl sulfate-polyacrylamide gel electrophoresis (SDS-PAGE) gel for protein size separation at a voltage of 100 V for 2 h. For CBB staining, the 10% CBB (Code No, C8430, Solarbio, Beijing, China) dissolved in acetic acid was used as the staining solution, and the acetic acid, ethanol, and sterile water (volume ration, 2:1:17) were used to destain the gel. CBB staining of the large rubisco subunit was treated as the loading control. For western blotting, whole proteins were transferred to nitrocellulose membranes (Hybond-C, GE Healthcare) as described previously [39]. Anti-GFP (Code No, D110008), and anti-FLAG (Code No, D110005) (Sangon Biotech BBI, Shanghai, China) antibodies were purchased. Anti-P1^SCSMV^ antibodies were prepared as described [39].

### Quantification of the relative expression levels of the target gene using real-time PCR

The CFX96 Touch Real-Time PCR Detection System (Bio-Rad, Hercules, USA) was used to quantify the relative expression levels of target genes using SsoFast EvaGreen Supermix (Bio-Rad) as previously described [5]. Bio-Rad CFX Manager 3.0 software was used to automatically calculate the cycle threshold (Ct) values, and the expression level of internal *EF1α* gene was used as an internal control for candidate gene expression analyses. The primers used for gene expression analysis are listed in **S1 Table**. All qRT-PCR was performed in triplicates. The values obtained were averaged.

### Identification of protein interactions

Yeast two-hybrid assay (Y2H), bimolecular fluorescent complementation assay (BiFC), and coimmunoprecipitation analyses (Co-IP) were performed to test the self-interaction of P1^SCSMV^. All these methods were performed as previously described [5].

For Y2H, the yeast GAL4 system was used to analyze the self-interaction of P1^SCSMV^. Different combinations of AD and BK plasmids was co-transformed into the AH109 strain. The transformants were plated on the SD/-Leu-Trp, SD/-Leu-Trp-His, SD/-Leu-Trp-His-Ade synthetic drop-out media with 10 mM 3-amino-1,2,4-triazole (Code No, A601149, Sangon Biotech BBI, Shanghai, China), and cultured for 5 days at 30°C. For BiFC, the binary plasmids that expressing of P1^SCSMV^ fusions (P1^SCSMV^ fused to the N-terminus of the C-terminal/N-terminal half of YFP) were constructed, and then these plasmids were combined and coinfiltrated into the leaves of *N*. *benthamiana*. After 3 dpi, the leaves were harvested and subjected to observation under confocal laser scanning microscopy at a wavelength of 512 nm by a Zeiss LSM-710 confocal microscope. For Co-IP analyses, the leaves that coexpressed the possible interaction combinations were collected and homogenized for total protein extraction. Then, the subsequent steps were as described previously [5]. The primers used are listed in the **S1 Table.**

### Preparation of DIG-labelled PVX-specific DNA probes and Northern blot assay

The coat protein (CP)-encoding sequences were labelled by PCR using digoxigenin-labelled dUTP (DIG-11-dUTP, Roche) as described previously [39]. Northern blot analyses were performed as previously described with minor modifications [39]. Briefly, 2 μg of total tissue RNA extracted from the leaves of *N*. *benthamiana* was separated by electrophoresis and transferred onto Hybond-N^+^ membranes (GE Healthcare, USA). DIG-AP-conjugated antibodies (Merck) were used to monitor PVX genomic RNAs. The detailed sequences of the probes are listed in **S2 Table.**

### Electrolyte leakage qualification

Plant stress responses result in electrolyte leakage from cells. Electrolyte leakage is widely used as a test for stress-induced injury of plant tissues as described previously with minor modifications [42]. To obtain the relative electrolyte leakage values of leaves upon different treatments, we first prepared a standard curve using the gradually increasing concentrations of NaCl (0, 10, 20, 30, 40, 60, 80, 100 μg/mL). The electrolyte leakage values were obtained by the measuring conductivity (model, DDS-11A, Shanghai LEICI, Shanghai, China) at 25°C. The infiltrated leaves were harvested and prepared as leaf discs with a diameter of 0.5 cm. For each treatment, five leaf discs were submerged in 20 mL of distilled water. A vacuum (model, RS-0.5, WOLIN, Zhengzhou, China) was used to extract air from the intercellular space, and the discs were completely infiltrated with distilled water. Electrolyte leakage was measured at 25°C. Each treatment was placed in boiling water for 10 min to kill all cells. After 10 min of cooling, the electrolyte leakage was measured using a conductivity meter. The obtained data were further processed, and the relative electrolyte leakage was determined.

### Trypan blue staining, 3,3’-diaminobenzidine tetrahydrochloride (DAB) staining, and tissue printing analyses

For trypan blue staining, the infiltrated leaves were cleaned with pure ethyl alcohol for 3 min, and then stained with trypan blue solution, followed by destaining with chloral hydrate, as previously described [43]. DAB staining was performed as previously described [44]. For tissue-printing analyses, the infiltrated leaves were sandwiched between two pieces of filter paper (Code No., 88610, Thermo Fisher Scientific, Shanghai, China), and pounded with a hammer. The filter paper was washed two times with sterile water containing 2% Triton X-100. Then, samples were processed as previously described [43].

### Determination of subcellular localization of the P1^SCSMV^ using the laser confocal microscopy

EGFP- or red fluorescent protein (RFP)-tagged P1^SCSMV^ at the C-terminus (P1^SCSMV^-EGFP and P1^SCSMV^-RFP) was overexpressed by *Agrobacterium*-mediated infiltration of leaves of *N*. *benthamiana*. Free RFP was also expressed in the nucleus and cytoplasm. After 3 dpi, leaves were collected and observed under a Zesis LSM710 confocal microscope, as previously described [5]. GFP and RFP were visualized at 488 nm and 543 nm, respectively, using an argon laser. The images were captured digitally with a Zeiss Axiocam camera in sequential scanning mode at a 1,024 x 1,024 pixel resolution, and processed by Imaris 7.4.2 software (Bitplane).

### RNA-immunoprecipitation assay

RNA-immunoprecipitation assay was performed as previously described [45]. In brief, the N-terminal 3 x Flag-tagged GUS, P1^SCSMV^, and P1^SCSMV^-nls were overexpressed via *Agrobacterium*-mediated infiltration of the leaves of *N*. *benthamiana*. At 3 dpi, leaves were harvested and ground using 3 volumes (w/v) of GTEN buffer with an additional recombinant RNase inhibitor (Code No., 2313Q, TaKaRa, Dalian, China), as described previously [5]. After centrifugation at 1000 x g for 30 min, Flag-GUS, Flag-P1^SCSMV^, and Flag-P1^SCSMV^-nls were purified using an anti-FLAG M2 affinity gel (Cat. No., A2220, Sigma-Aldrich, Shanghai, China). Target protein-bound RNAs were extracted by TRIzol Reagent (Cat. No., 15596018, Thermo Fisher Scientific, Shanghai, China). The specific pairs of primers used for quantify *NbbZIP60U* and *NbbZIP60S* by qRT-PCR are listed i**n S1 Table.**

### Protein expression and purification from the *E*. *coli*

The N-terminal glutathione S-transferase-tagged P1^SCSMV^ and mutant P1^SCSMV^-nls (GST-P1^SCSMV^ and GST-P1^SCSMV^-nls) were expressed in *E*. *coli* strain BL21 (DE3) pLysS cells (Novagen) as described previously with minor modifications [5]. Recombinant protein expression was induced by the addition of 100 μM isopropyl β-D-1-thiogalactopyranoside (IPTG, Sigma-Aldrich) at a rotation speed of 220 r/min for 12 h at 18°C. The GST-tagged recombinant protein purified as previously described [46]. Briefly, the cells were harvested and suspended in buffer T (50 mM Tris-HCl, pH 9.0, 500 mM NaCl, 10% glycerol,1 mM PMSF), and disrupted by the ultrasonication (Model 500 Homogenizer, Fisher Scientific, Pittsburgh, USA) for 25 min at 250 W, 20 kHz, and 2 s working/1 s interval, followed by centrifugation at 16,000 X g for 25 min at 4°C. The supernatant was passed through a Glutathione-Sepharose affinity column (GE Healthcare, Little Chalfont, UK). Sepharose-bound recombinant proteins were eluted using T-buffer containing 60 mM L-glutathione and 2 mM DTT. Similarly, the recombinant proteins were further concentrated by an Amicon Ultra-15 filter unit (Millipore), and stored in a -80°C refrigerator after repackaging. The primers used are listed in **S1 Table.**

### Electrophoretic mobility shifts assay (EMSA)

EMSAs were performed as previously described with minor modifications [43]. To prepare the RNA probe, a 532-bp 5’-biotinylated RNA of partial *NbbZIP60U* mRNA containing the stem-loop region and its mutant (**S2 and S3 Tables**) was transcribed by the T7 RNA polymerase *in vitro* and recovered by the DNase I treatment, phenol-chloroform extraction, and ethanol precipitation. In brief, 532-bp cDNA from *NbbZIP60U* mRNA was cloned into the pSPT19 (Roche) plasmid. To start *in vitro* transcription, linearized pSPT19-*NbbZIP60U/P* with *BsrG* I digestion was treated as a template, and the substrate that contained 2.5 mM of ATP, CTP, GTP (Sangon, Shanghai, China), and biotin-16-UTP (SKU,11388908910, Roche, Merck, Shanghai, China). The transcribed *NbbZIP60U* probe was labeled with biotin and quantified using a NanoDrop ND-1000 spectrophotometer (Nanodrop Technologies, DE, USA). Furthermore, we mutated the stem-loop region of the pSPT19-*NbbZIP60U/P* vector to generate the *NbbZIP60U-M* probe.

Purified GST-P1^SCSMV^ and P1^SCSMV^-nls were incubated with 532-bp RNA probe at equal molar ratios at 25°C for 30 min. The protein-RNA complex was run on a nondenaturing agarose gels in 0.5 x TBE buffer for 1 h at 60 V at 4°C and then transferred to a Hybond N^+^ nylon membrane (GE Healthcare) as described previously [46]. Mobility shifts of biotin-labelled RNAs were detected using streptavidin-horseradish peroxidase (HRP) conjugates (Cat No., SA00001-0, Proteintech Group, Rosemont, USA). Signals were visualised using reagents included in the kit and ChemiDoc XRS (Bio-Rad Laboratories, UAS).

### Statistical analysis

Student’s *t-test* was performed to determine statistical significances in UPR activation and inhibition analyses. Other data were subjected to statistical analysis using SPSS software (version 22.0, IBM). Comparison was performed using one-way analysis of variance (ANOVA). Significant differences were determined using Duncan’s multiple range test. ImageJ software (v2.0, National Institutes of Health, Bethesda, MD, USA, http://imagej.nih.gov/ij/, accessed on 9 September 2022) was used to quantify the intensity of the RT-PCR bands as previous described [39].

## Results

### P1 of Sugarcane streak mosaic virus acts as a classical viral-encoded gene silencing suppressor

**Fig 1:**
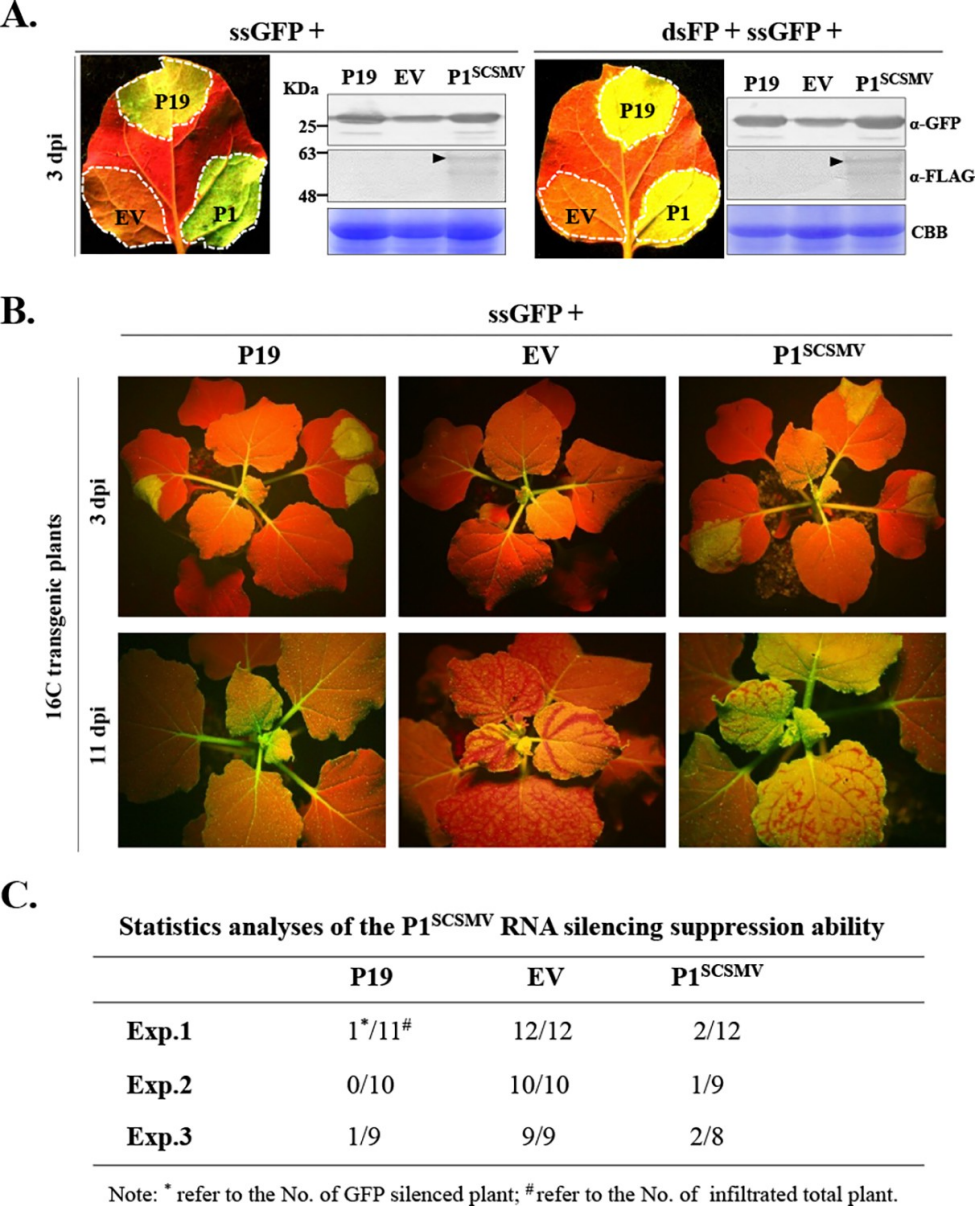
Sugarcane streak mosaic virus P1 (P1^SCSMV^) suppresses both local and systemic RNA silencing in *Nicotiana benthamiana*. **(A).** P1^SCSMV^ could suppress the single-strand sense-GFP (ssGFP) induced local RNA silencing with and without the C-terminal of double-strand GFP fragment (dsFP). The p19 from tomato bushy stunt virus was used as positive control, EV means empty vector. At 3 dpi, the leaves were photographed under a UV lamp, and the proteins were detected with the commercial anti-GFP and anti-FLAG antibodies. Black arrow indicates the target proteins. **(B).** P1^SCSMV^ could suppress the single-strand sense-GFP (ssGFP) induced systemic RNA silencing. The infiltrated leaves were photographed under a UV lamp after 3 dpi and 11 dpi. **(C).** Statistical analyses of the systemic RNA silencing suppression ability of P1^SCSMV^.

RNA silencing is an evolutionarily-conserved and sequence-specific gene inactivation system. RNA silencing is a major mechanism used to defend against viruses in plants and insects [47]. Sugarcane streak mosaic virus (SCSMV) belongs to the same *genus* as *triticum* mosaic virus. P1 of SCSMV (P1^SCSMV^), was previously demonstrated to suppress local RNA silencing in 16c transgenic *Nicotiana benthamiana* [36]. However, the RNA-silencing suppression (RSS) activity of the P1^SCSMV^ were not explored in a systematic and comprehensive manner. We performed an agro-infiltration assay to analyze the RSS activity of P1^SCSMV^. *Agrobacteria* transformed with the binary plasmid pGD-ssGFP were mixed with an equal volume of another *A*. *tumefaciens* suspension harbouring the empty vector pGWB5 (EV, negative control), pGWB5-P1^SCSMV^, or pGWB5-p19 (positive control) plasmid, and then infiltrated into three different areas of a *N*. *benthamiana* leaf. At 3-day-post-infiltration (dpi), the leaves were harvested and examined under a longwave ultraviolet lamp. P1^SCSMV^-infiltrated area emitted strong green fluorescence, and the accumulation levels of green fluorescence protein (GFP) were higher than those in the EV-infiltrated area, as well as in the positive control of tomato bushy stunt virus p19 (**Fig 1A, left panel**). Similarly, we also used an agrobacteria mixture transformed with pGD-dsFP and pGD-ssGFP instead of a single agrobacterium harbouring the plasmid pGD-ssGFP and performed the same agro-infiltration assay. Same results were observed (**Fig 1A, right panel**). Hence, P1^SCSMV^ was found to suppress both ssGFP and dsFP (partial coding sequences of the GFP) induced local RNA silencing.

The ability of P1^SCSMV^ to suppress systemic RNA silencing was analysed using 16C transgenic *N*. *benthamiana*. 3 dpi, local leaves infiltrated with P1^SCSMV^- and p19 emitted more intense green fluorescence than the EV-infiltrated leaves (**Fig 1B, upper panel**). At 11 dpi, systemic leaves of the P1^SCSMV^- and p19-infiltrated plants also showed more intense green fluorescence than that emitted by the systemic leaves of the EV-treated plants (**Fig 1B, bottom panel**). Three independent experiments were performed to illustrate the systemic RNA silencing suppression ability of P1^SCSMV^. Statistical data analysis revealed that the most systemic leaves of P1^SCSMV^- and p19-infiltrated plants exhibited intense green fluorescence, indicating that P1^SCSMV^, efficiently suppression of systemic *GFP* silencing (**Fig 1C**). Taken together, these results demonstrate that P1^SCSMV^ functions as a classical VSR through suppression of both local and systemic RNA silencing in *N*. *benthamiana*.

### Heterologous expression of P1^SCSMV^ enhances potato virus X infection and induces plant cell death in *N*. *benthamiana*

**Fig 2:**
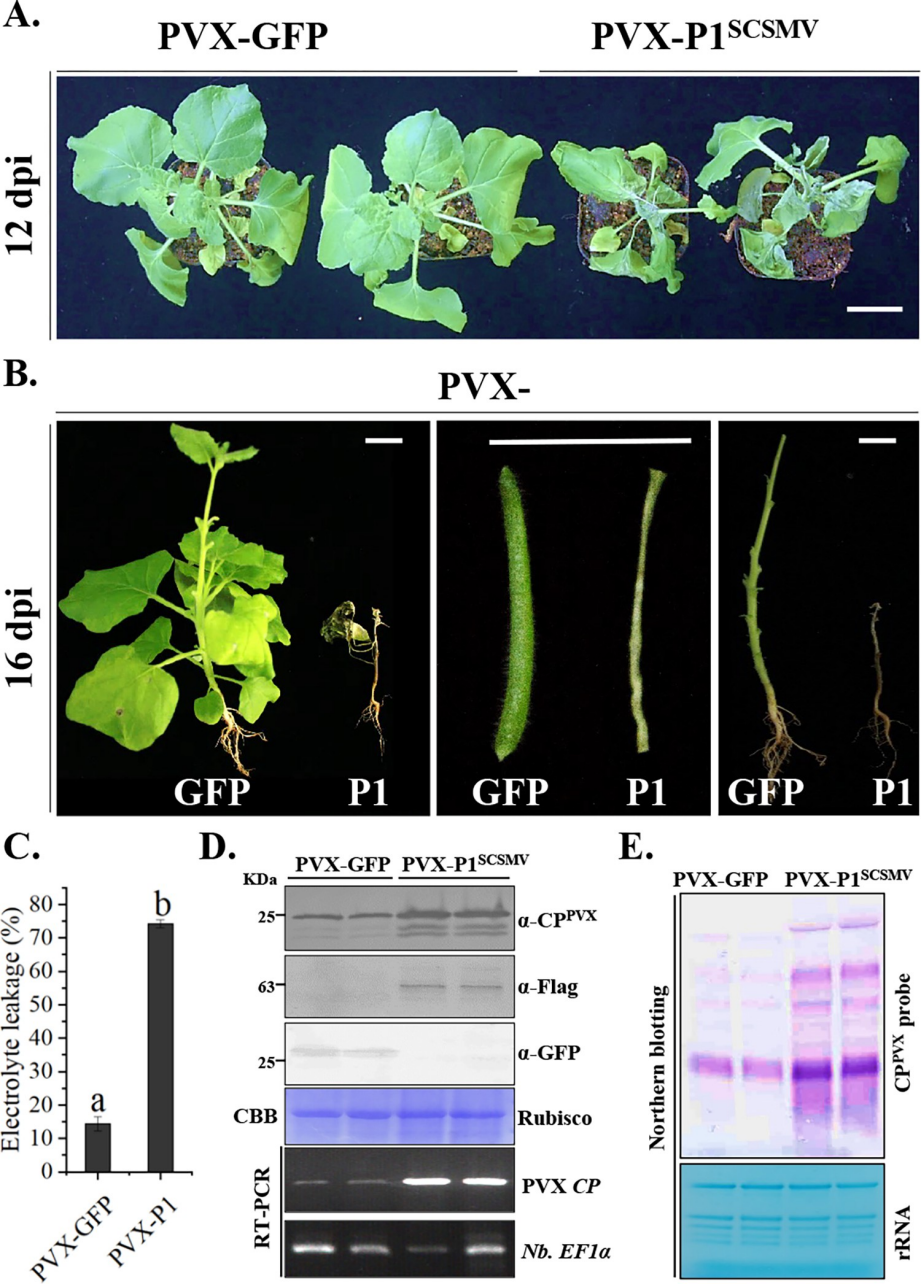
P1^SCSMV^ promotes the pathogenicity of the PVX-derived recombinant virus infection in *N*. *benthamiana*. **(A).** Disease of the PVX-GFP and PVX-P1^SCSMV^ infected *N*. *benthamiana* at 12 dpi. bar represents 3 cm. **(B).** The disease appeared in all parts of the plant, petiole, and culm upon recombinant PVX-P1^SCSMV^ infection at 16 dpi. bar represents 3 cm. **(C).** The intensity of cell-death in the local leaves were quantified by the relative electrolyte leakage at 8 dpi. “a” and “b” means significant difference between these two columns. **(D).** Western blot and RT-PCR confirmed the recombinant PVX-GFP and PVX-P1^SCSMV^ infection at 8 dpi. Similarly, commercial anti-GFP and anti-FLAG antibodies, and anti-CP^PVX^ were used for detection of the expressed GFP, P1^SCSMV^, and CP^PVX^. The PVX *CP* coding sequences was used for quantification of the recombinant PVX accumulation. **(E).** Northern blot detection of viral genomic RNA accumulation in recombinant virus PVX-GFP and PVX-P1^SCSMV^ infected condition at 12 dpi. The ribosome RNAs (rRNA) were stained as loading control.

To assess the pathogenicity of P1^SCSMV^, we overexpressed N-terminal Flag-tagged P1^SCSMV^ using potato virus X vector pND108 [48,49]. Agrobacteria harboring pND108-P1^SCSMV^ or pND108-GFP were infiltrated into 8-leaf-stage *N*. *benthamiana* plants. The systemic leaves of P1^SCSMV^ infiltrated plants showed chlorotic and mild mosaic spots at 6 dpi, followed by mild rolling at 9 dpi. The pND108-P1^SCSMV^-infiltrated leaves showed mild cell-death at 6 dpi, whereas the pND108-GFP-infiltrated leaves did not (**Fig 2A**). At 12 dpi, the leaves of pND108-P1^SCSMV^-infiltrated plants displayed severe cell death, and abnormal development of apical lobes (**Fig 2A**), compared to mosaic and chlorotic symptoms in pND108-GFP-infiltrated plants (**Fig 2A**). Systemic leaves of *Agrobacteria* infiltrated plants were harvested, and subjected to cell death analyses by measuring electrolyte leakage at 8 dpi (**Fig 2C**). The results showed that the cell death intensity of systemic leaves from pND108-P1^SCSMV^-infiltrated plants were more severe than that of systemic leaves from pND108-GFP-infiltrated plants, which indicating the increased pathogenicity of P1^SCSMV^ to PVX (**Fig 2C**). Protein immunoblotting analysis indicated expression of CP^PVX^, Flag-P1^SCSMV^, and GFP in recombinant PVX-infected plants. Systemic leaves infected with PVX-P1^SCSMV^ displayed higher levels of CP^PVX^ than those infected with PVX-GFP, demonstrating enhanced PVX infection by P1^SCSMV^ (**Fig 2D, upper panel**). The RT-PCR results also showed higher levels of transcripts of *CP* gene of PVX (*CP*^*PVX*^) in systemic leaves of pND108-P1^SCSMV^-infiltrated plants compared to these in pND108-GFP-infiltrated plants, indicating enhanced pathogenicity of the PVX by P1^SCSMV^ (**Fig 2D, bottom panel**). Furthermore, northern blot results showed higher levels of PVX genomic RNAs in systemic leaves of pND108-P1^SCSMV^-infiltrated plants than in the systemic leaves of pND108-GFP-infiltrated plants using the digoxin-labelled *CP*^*PVX*^ probe, which also demonstrated that P1^SCSMV^ enhanced PVX infection (**Fig 2E**). At 16 dpi, pND108-P1^SCSMV^-infiltrated plants fully exhibited systemic cell death, whereas the pND108-GFP-infiltrated plants did not (**Fig 2B**). In summary, all findings indicate that P1^SCSMV^ promotes PVX infection and induces cell death in *N*. *benthamiana*.

### P1^SCSMV^ inhibited the ER stress-induced unfolded protein response

**Fig 3:**
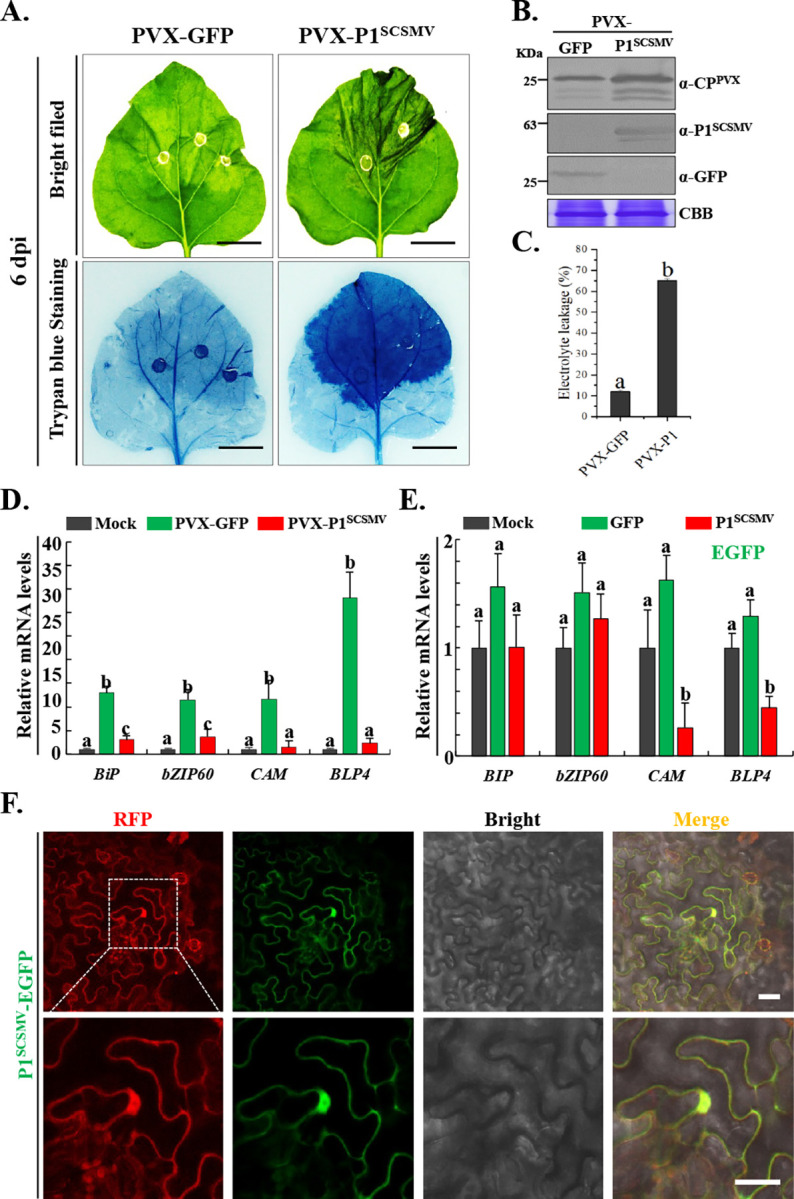
P1^SCSMV^ suppresses the expression of IRE1/bZIP60 marker genes in unfolded protein response (UPR) signaling pathway. **(A).** PVX-GFP and PVX-P1^SCSMV^ infiltrated leaves were photographed before and after trypan blue staining at 6 dpi. bar represents 2 cm. **(B).** Western blot confirmed the expression of the CP^PVX^, GFP, and P1^SCSMV^ using commercial and self-prepared antibodies at 6 dpi. **(C).** The cell-death intensity of the systemic leaves was quantified by the relative electrolyte leakage at 6 dpi. **(D).** The relative expression levels of IRE1/bZIP60 UPR marker genes were determined at 6 dpi. Different letters (a, b, and c) mean significant difference between these three columns which represent the mock-treated, PVX-GFP-infected, and PVX-P1^SCSMV^-infected conditions. **(E).** The relative expression levels of the marker genes of IRE1/bZIP60 UPR signaling pathway were quantified at 6 dpi. The three columns in each gene represent mock-treated, transient expression of GFP, and transient expression of P1^SCSMV^, respectively. **(F).** Determination of the subcellular localization of the P1^SCSMV^ at 3 dpi. The white frame is enlarged and is listed in the bottom channel. bar represents 50 μm.

To further determine whether cell death was caused by the overexpression of P1^SCSMV^ in *N*. *benthamiana*, we first compared the cell death levels between pND108-P1^SCSMV^ and pND108-GFP-infiltrated leaves at 6 dpi using trypan blue staining. PVX-P1^SCSMV^-infected local leaves were found to be more severely infected than the PVX-GFP-infected local leaves (**Fig 3A**). Western blotting revealed GFP and P1^SCSMV^ expression in local leaves, with PVX-P1^SCSMV^ plants displayed higher levels of CP^PVX^ in the local leaves at 6 dpi, indicating that P1^SCSMV^ enhances PVX pathogenicity (**Fig 3B**). Electrolyte leakage from PVX-P1^SCSMV^-infected local leaves was higher than that from PVX-GFP-infected local leave at 6 dpi (**Fig 3C**), indicating that P1^SCSMV^ could cause cell death in the presence of PVX infection. Endoplasmic reticulum (ER)-dependent replication of PVX causes an ER stress- induced unfolded protein response (UPR). The triple-gene-block 1 and 2 proteins (pTGB1 and pTGB2) are the major inducers of UPR. Hence, we next investigated whether P1^SCSMV^ affects the PVX infection-induced UPR. For this purpose, we selected four candidate marker genes, *BiP*, *bZIP60*, *CAM*, and *BLP4*, and quantified their relative expression levels following PVX-GFP and PVX-P1^SCSMV^ infection using qRT-PCR at 5 dpi. Here, we used a pair of primers, bZIP60/F & bZIP60/S/F, to quantify the total *bZIP60S* transcripts (spliced form) in PVX recombinant viruses’ infection. Four UPR marker genes were found to be upregulated in PVX-GFP-infected leaves, whereas only *BiP* and *bZIP60* were significantly down-regulated in PVX- P1^SCSMV^-infected leaves compared to PVX-GFP-infected leaves (**Fig 3D**). Thus, P1^SCSMV^ suppressed the UPR signaling pathway. To further determine the role of P1^SCSMV^ in inhibition of UPR signaling, we transiently expressed GFP and P1^SCSMV^ via *Agrobacteria*-mediated infiltration. We measured the cell death intensity and the expression levels of the four UPR marker genes in the infiltrated leaves at 3 dpi. The qRT-PCR results showed that only *CAM* and *BLP4* were downregulated in P1^SCSMV^ transient-expressing leaves compared to mock-treated or GFP-expressing leaves (**Fig 3E**). These results confirmed suppression of UPR signaling pathway by P1^SCSMV^, with the downstream functional genes responsible for ER-stress alleviation being particularly affected.

To clarify the subcellular location of P1^SCSMV^ function, we overexpressed the C-terminal EGFP-tagged P1^SCSMV^via *Agrobacteria*-mediated infiltration. The infiltrated leaves were collected and observed under a confocal microscope at 3 dpi. P1^SCSMV^ was found to be localized to both the nucleus and cytoplasm. Free EGFP served as a control for the nuclear and cytoplasmic localization markers (**Fig 3F**). The UPR is known to take place mainly on the ER membrane. Thus, the observed UPR suppression may be linked to the cytoplasmic localization of P1^SCSMV^, especially when the ER is distributed in the cytoplasm and is hardly distinguished under confocal microscopy without a ER marker.

### Bipartite basic regions (BMs) determined the nuclear localization of the P1^SCSMV^

**Fig 4:**
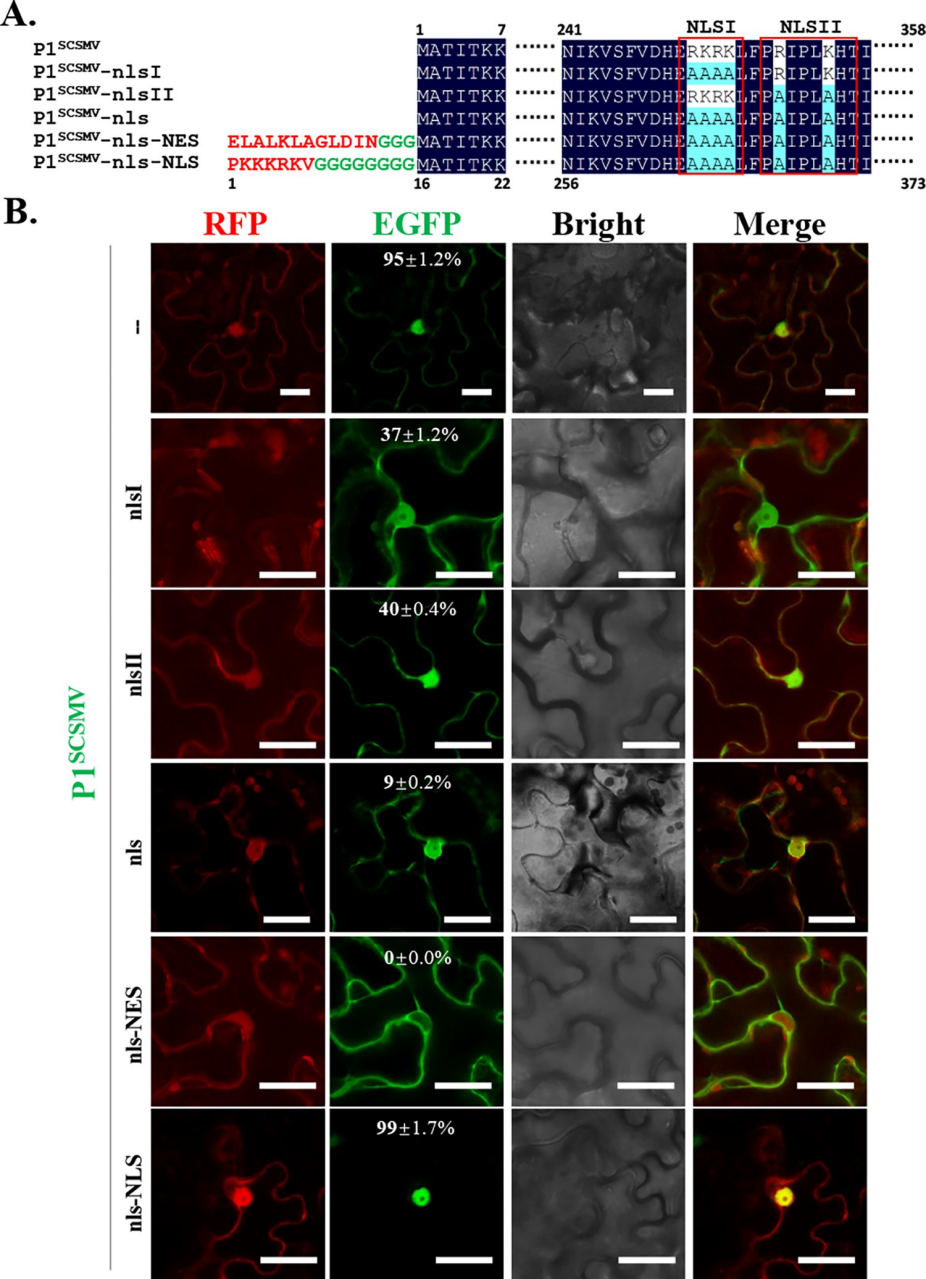
P1^SCSMV^ has a C-terminal bipartite nuclear localization signals (NLS). **(A).** Constructs Illustration of different GFP-tagged P1^SCSMV^. Red letters indicate the C-terminal conventional nuclear export signal (NES) peptide and a N-terminal conventional NLS in the mutants P1^SCSMV^-nls-NES and P1^SCSMV^-nls-NLS, respectively. Green letters represent the linker between the signal peptide and the start amino acid (aa, Met) of P1^SCSMV^. The marked numbers indicate the aa positions. Red frames indicate the two NLS. **(B).** Subcellular localization of different P1^SCSMV^ mutants listed above. Free red fluorescence protein (RFP) was co-expressed with GFP-tagged P1^SCSMV^ and its derivatives. After 2 dpi, leaves were harvested and observed.

To explore the possible elements that determine the subcellular localization of P1^SCSMV^, we predicted nucleus localization signal (NLS) or other signal peptides using software or platforms available online (https://nls-mapper.iab.keio.ac.jp/cgi-bin/NLS_Mapper_form.cgi) (**S1A Fig**). The predictions showed that P1^SCSMV^ has a possible bipartite NLS across basic motifs (BM) from amino acids (aa) 251 to 254 (BM1) and 257 to 263 (BM2) (**Fig 4A**). We mutated the basic aa in BM1 and BM2 to alanine (A) and generated P1^SCSMV^-nlsI, P1^SCSMV^-nlsII, and P1^SCSMV^-nls to further analyzed the subcellular localization of P1^SCSMV^. We also constructed two P1^SCSMV^ mutants based on the P1^SCSMV^-nls, which included an N-terminal conventional NLS peptide-tagged P1^SCSMV^ (P1^SCSMV^-nls-NLS, positive control) and a C-terminal conventional nuclear export signal (NES) peptide-tagged P1^SCSMV^ (P1^SCSMV^-nls-NLS, negative control) (**Fig 4A**). All P1^SCSMV^ mutants were fused to the N-terminus of EGFP, and co-expressed with free RFP in the epidermal cells of *N*. *benthamiana*. After 3 dpi, the leaves were collected and the target protein’s expression were confirmed by western blot (**S2 Fig**). The number of cells localized to the nucleus of each P1^SCSMV^ mutants were counted. The nuclear localization ratios of the different P1^SCSMV^ mutants were also calculated (**Fig 4B**). Accordingly, most of the wild-type P1^SCSMV^ (95%) localized was localized to the nucleus, whereas less than half of P1^SCSMV^-nlsI and P1^SCSMV^-nlsII was in the nucleus (**Fig 4B, upper**). When both BM1 and BM2 were mutated, the subcellular localization ratio decreased to 9%. In other words, the majority of the double BMs mutant of P1^SCSMV^ (P1^SCSMV^-nls) lost their nuclear localization ability (**Fig 4B, bottom**). These results demonstrated that both BM1 and BM2 are required for P1^SCSMV^ nuclear localization, and they are the NLS of P1^SCSMV^.

### NLS of P1^SCSMV^ are required for self-interaction *in vitro* and *in vivo*

**Fig 5:**
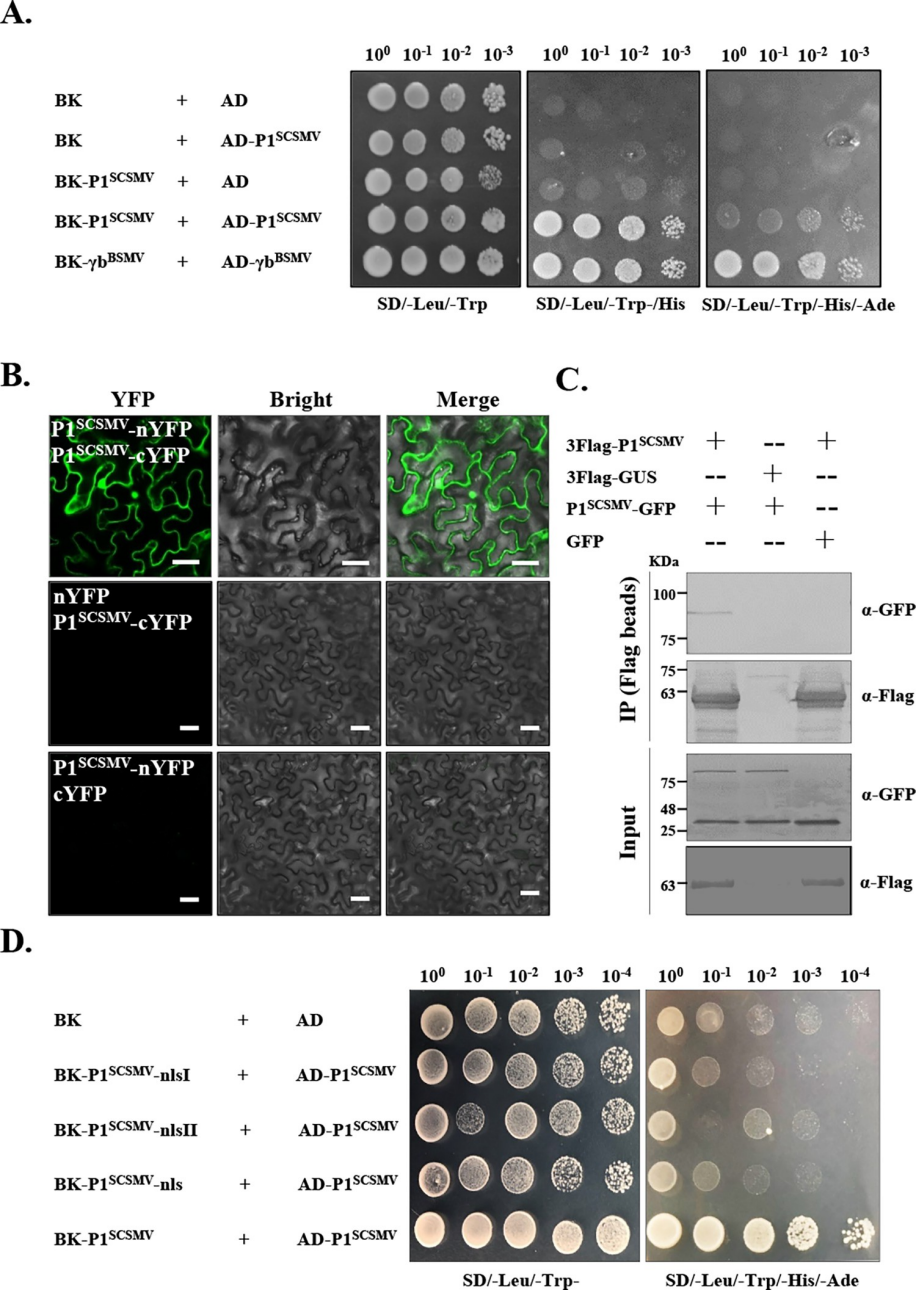
Self-interactions of P1^SCSMV^ and its NLS mutants *in vivo* and *in vitro*. **(A).** P1^SCSMV^ was found to interact with itself in the yeast two-hybrid assay (Y2H). γb protein of barley stripe mosaic virus (BSMV) served as the positive control for the self-interaction. Top number indicate the dilution folds of the yeast cells plated in different type of nutrient deficient medium. **(B).** P1^SCSMV^ associated with itself *in vivo* in the bimolecular fluorescence complementary assay (BiFC). YFP signals were emitted under a 512 nm wave-length laser and depicted as a false-green color. Scale bar, 40 μm. **(C).** P1^SCSMV^ was found to interact with itself *in vivo* in co-immunoprecipitation (Co-IP). The plus sign in each lane indicates co-expression of the two proteins. The commercial anti-GFP and anti-FLAG antibodies were used to confirm the presence of the target protein. **(D).** The effects of the NLS on P1^SCSMV^ to the self-interaction ability were determined. The self-interaction of the P1^SCSMV^ served as the positive control.

Most VSRs of plant viruses, including 2b, p19, Hc-Pro, and γb, are often form dimers and bind the siRNA to inhibit host antiviral RNA silencing. To clarify whether the P1^SCSMV^ also interacts with itself, we first used a *gal4*-based yeast two-hybrid (Y2H) system to test the self-interaction of P1^SCSMV^. The results showed that only BK-P1^SCSMV^ + AD-P1^SCSMV^ co-transformed yeast cells grew significantly on the SD/-Leu-Trp and SD/-Leu-Trp-His-Ade drop-out medium with the addition of 3-AT, as well as the positive control of AD-γb^BSMV^ + BK-γb^BSMV^ co-transformed yeast (**Fig 5A**). Furthermore, a bimolecular fluorescence complementation assay (BiFC) also showed that the N-terminal half of the YFP-tagged P1^SCSMV^ (P1^SCSMV^-nYFP), together with the C-terminal half of the YFP-tagged P1 ^SCSMV^ (P1^SCSMV^-cYFP), formed a complete YFP protein *in vivo* and emitted intense YFP signals under a 512 nm length laser (**Fig 5B**). Hence, P1^SCSMV^ associates with itself *in vivo*. Moreover, coimmunoprecipitation (Co-IP) analyses further showed that only P1^SCSMV^-GFP could be precipitated using Flag-P1^SCSMV^ with commercial flag beads, whereas free GFP could not be precipitated (**Fig 5C**), indicating self-interaction *in vivo* as well. In summary, these results clearly demonstrated that P1^SCSMV^ physically interacts with itself *in vivo* and *in vitro*.

To clarify the effects of the identified NLS on P1^SCSMV^ self-interaction, we performed a Y2H assay (**Fig 5D**). NLS mutants, including the P1^SCSMV^-nlsI, P1^SCSMV^-nlsII, and P1^SCSMV^-nls, were fused to the BK to generate mutant BK vectors. Each type of BK-fused protein, together with the AD-P1^SCSMV^, was expressed in yeast cells (**S5 Fig**). None of the combinations resulted in growth in the SD/-Leu-Trp-His-Ade drop-out medium with the addition of 3-AT, except for the positive control (AD-γb^BSMV^ + BK-γb^BSMV^) (**Fig 5D**), and there is barely visible YFP signal under confocal observation *in vivo* (**S7 Fig**), which demonstrated that the bipartite NLS peptides are required for the self-interaction of P1^SCSMV^.

### Nucleocytoplasmic shuttling of P1^SCSMV^ is essential for sustaining the RSS activity

**Fig 6:**
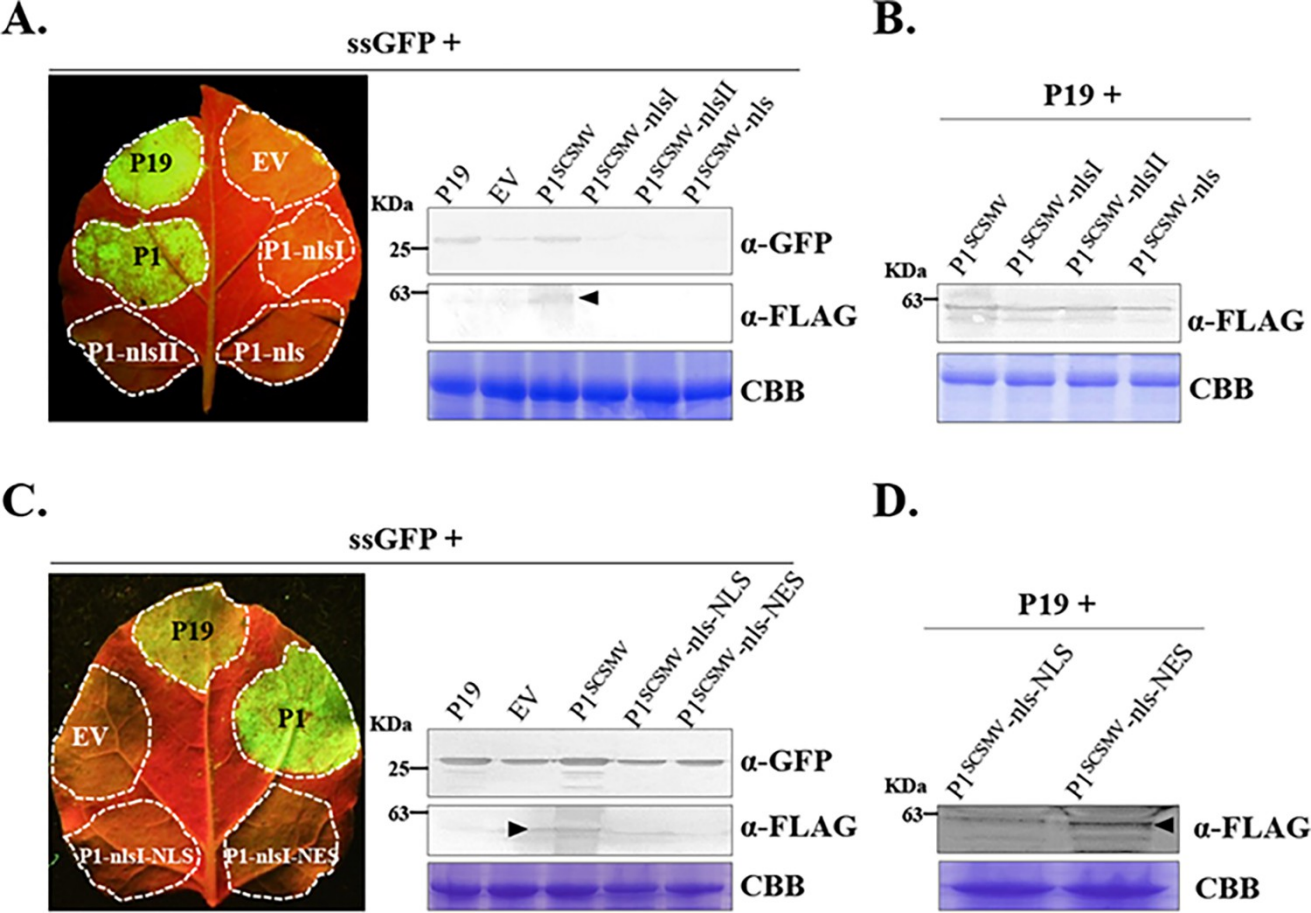
RNA silencing suppression (RSS) activity of P1^SCSMV^ and its NLS-related mutants. **(A).** RSS activity of P1^SCSMV^ and its NLS mutants. The leaves were collected at 3 dpi. Black arrow indicates the target protein. The commercial anti-GFP and anti-FLAG antibodies were used to confirm the expression of the target protein. **(B).** Western blot detected the P1^SCSMV^-NLS mutants that were not detected in RSS activity assay. **(C).** RSS activity of P1^SCSMV^ and its compelling-nuclear-importing (P1^SCSMV^-NLS) and compelling-nuclear-exporting mutant (P1^SCSMV^-NES). **(D).** Western blot detection of protein expression of these P1^SCSMV^ mutants.

To determine whether the NLS peptides of P1^SCSMV^ affect the RSS activity, we next performed a ssGFP-spot experiment by overexpressing of the single-strand sense GFP (ssGFP) and P1^SCSMV^ or their different mutations through *Agrobacterium*-mediated infiltration on *N*. *benthamiana*. Only the wild-type P1^SCSMV^-infiltrated area was found to emit green fluorescence under a longwave ultraviolet lamp, similar to the positive control (p19-infiltrated area). All other NLS mutation-expressing areas displayed barely invisible green fluorescence (**Fig 6A, left**). A protein immunoblot assay confirmed these results using GFP-specific antibodies (**Fig 6A, right**). Western blot also validated that P1^SCSMV^ and all its NLS mutations were expressed in the infiltrated area by using the antibodies against the Flag tag (**Fig 6B**). These results indicated that the NLS elements of P1^SCSMV^ are essential for sustaining the RSS activity.

To clarify whether the nucleocytoplasmic shuttling of P1^SCSMV^ is required to sustain RSS activity, we further detected the RSS activity of P1^SCSMV^ mutants that remained in the cytoplasm (P1^SCSMV^-nls-NES) or nucleus (P1^SCSMV^-nls-NLS). Neither the P1^SCSMV^-nls-NES nor P1^SCSMV^-nls-NLS expressed area was found to display visible green fluorescence, which indicated that neither complete nucleus localization nor cytoplasmic localization of P1^SCSMV^ is related to its RSS activity (**Fig 6C, left**). Western blotting showed that the GFP accumulation levels were consistent with our observations (**Fig 6C, right**). Western blotting revealed that all proteins were expressed in the infiltrated area using commercial anti-FLAG antibodies (**Figs 6C, 6D, and S2**). These results indicated that uniform distribution of the nucleus and cytoplasm of P1^SCSMV^ is essential for RSS activity display. In summary, nucleocytoplasmic shuttling of P1^SCSMV^ is essential for sustaining RSS activity.

### Nucleocytoplasmic shuttling of P1^SCSMV^ is required for the PVX pathogenicity and UPR inhibition

**Fig 7:**
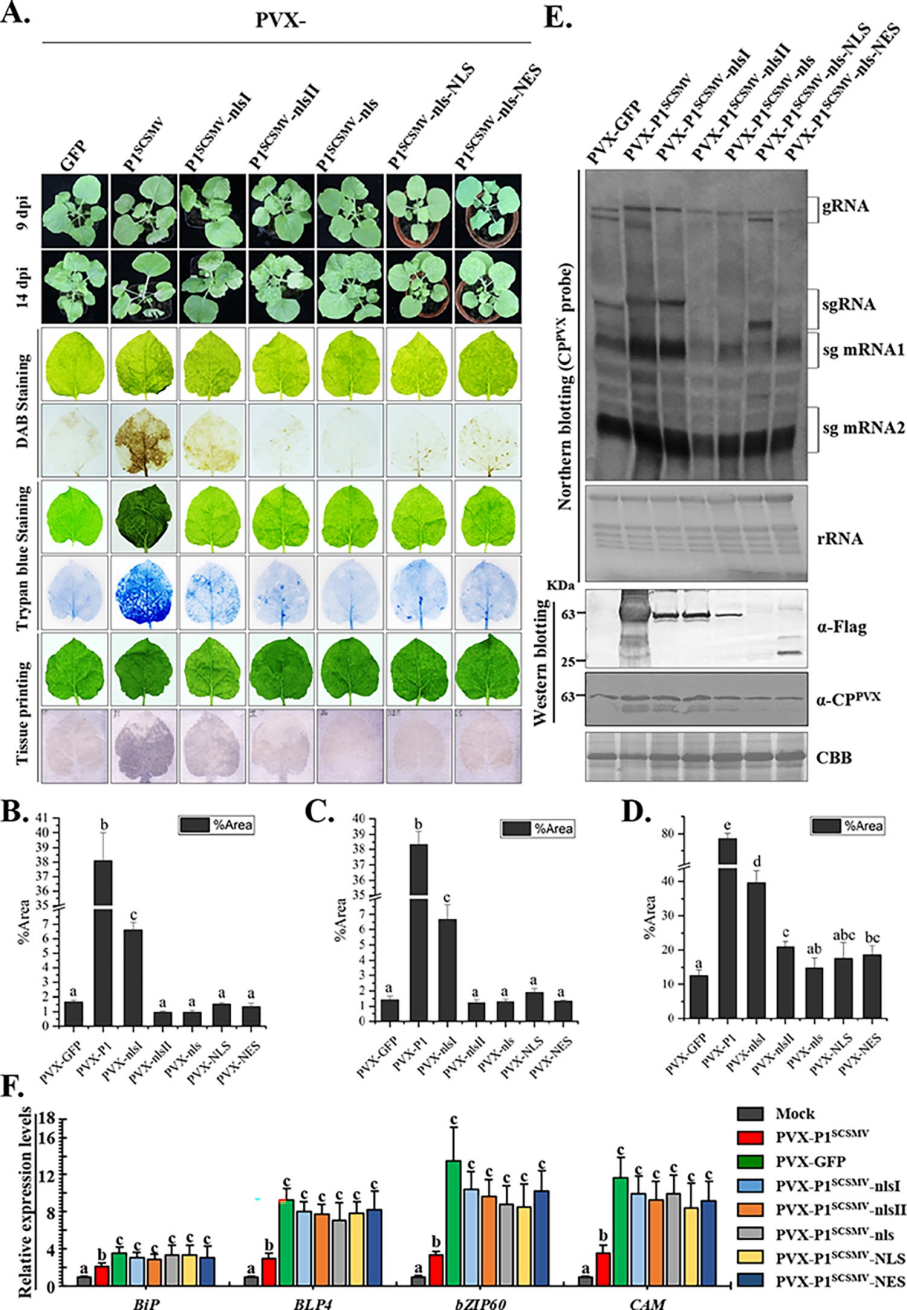
Effects on the recombinant PVX pathogenicity and IRE1/bZIP60 UPR signaling pathway suppression ability of P1^SCSMV^ and its NLS-related mutants. **(A).** Symptoms of the recombinant virus by *cis-* heterologous expressing of the P1^SCSMV^ and its NLS-related mutants through PVX at 9 dpi and 14 dpi. The virus-infected systemic leaves at 9 dpi were harvested and subjected to 3,3’-diaminobenzidine tetrahydrochloride (DAB) staining, trypan blue staining, and tissue printing analyses. **(B)-(D)**. Relative intensity of the ROS, cell-death, and virus accumulation were quantified by the ImageJ software. Different letters (a, b, and c, et. al.) mean significant difference between these columns. **(E).** Northern blot (upper panel) and western blot were used to determine (bottom panel) the viral genomic RNA and P1 mutant’s accumulations in recombinant viruses infected condition at 9 dpi. The antibodies, genomic RNA, and sub-genomic RNA bands are marked on the right side of the images. **(F).** Relative expression levels of marker genes in IRE1/bZIP60 UPR signaling pathway under recombinant PVX viruses’ infection. Different letters (a, b, and c, et. al.) mean significant difference between these columns as described above.

To further explore the effect of nucleocytoplasmic shuttling of P1^SCSMV^ on PVX pathogenicity and the UPR signaling pathway, we overexpressed NLS mutants of P1^SCSMV^ via recombinant PVX through *Agrobacterium*-mediated infiltration of *N*. *benthamiana*. At 9 dpi, the PVX-P1^SCSMV^-infiltrated leaves died, whereas the other recombinant virus-inoculated leaves did not (**Fig 7A, upper**). After 14 dpi, the upper leaves of the PVX-P1^SCSMV^-infected plants exhibited a severe cell death phenotype, whereas the other recombinant virus-infected systemic leaves did not (**Fig 7A, upper**). Furthermore, we also collected systemic leaves from each recombinant virus-infected plant, and subjected them to DAB, trypan blue, and tissue staining. The leaves were photographed before and after staining (**Fig 7A, bottom**). DAB staining was used for *in situ* detection of hydrogen peroxide in virus-infected systemic leaves [50]. Trypan blue staining and tissue printing are often used to measure the cell death intensity and virus accumulation, respectively [43]. The staining results showed that reactive oxygen species (ROS), cell death intensity, and PVX accumulation levels were higher in the systemic leaves of PVX-P1^SCSMV^-infected plants than in the upper leaves of PVX recombinant virus-infected plants (**Fig 7A, bottom**). ImageJ software was used to quantify the relative intensity of ROS, cell death, and virus accumulation in the displayed images [51]. These results agree with the photographs (**Figs 7B, 7C, and 7D**), which indicated that the nucleocytoplasmic shuttling of P1^SCSMV^ contributed to ROS generation, cell death, and PVX accumulation.

To further determine the effect of the nucleocytoplasmic shuttling of P1^SCSMV^ on virus accumulation, we performed northern blot analyses using a digoxin-labelled *CP*^*PVX*^ probe. PVX-P1^SCSMV^-infected plants were found to have a higher genomic RNA accumulation level than those found in other P1^SCSMV^ NLS mutant recombinant viruses (**Fig 7E, upper panel**), indicating that nucleocytoplasmic shuttling of P1^SCSMV^ promoted PVX accumulation. Western blot analyses further demonstrated that all P1^SCSMV^ NLS mutants were expressed in virus-infected plants using specific anti-FLAG antibodies. PVX-P1^SCSMV^-infected plants were found to accumulated highest CP^PVX^ compared to other recombinant PVX-infected plants that expressed different P1^SCSMV^ NLS mutants. This further demonstrates that the NLS was essential for the pathogenicity of P1^SCSMV^ (**Fig 7E, bottom**). Moreover, we explored the effects of nucleocytoplasmic shuttling of P1^SCSMV^ on the UPR signaling pathway. The qRT-PCR results showed that the relative expression levels of *BiP*, *bZIP60*, *CAM*, and *BLP4* in PVX-P1^SCSMV^-derived recombinant virus-infected plants were lower than those in PVX-GFP-infected plants (**Fig 7F**), indicating that P1^SCSMV^ partially inhibited the UPR signaling pathway under PVX infection conditions.

### Artificial inhibition of the nuclear shuffling of P1^SCSMV^ and IRE1-bZIP60-associated UPR signaling pathways promoted cell-death

**Fig 8:**
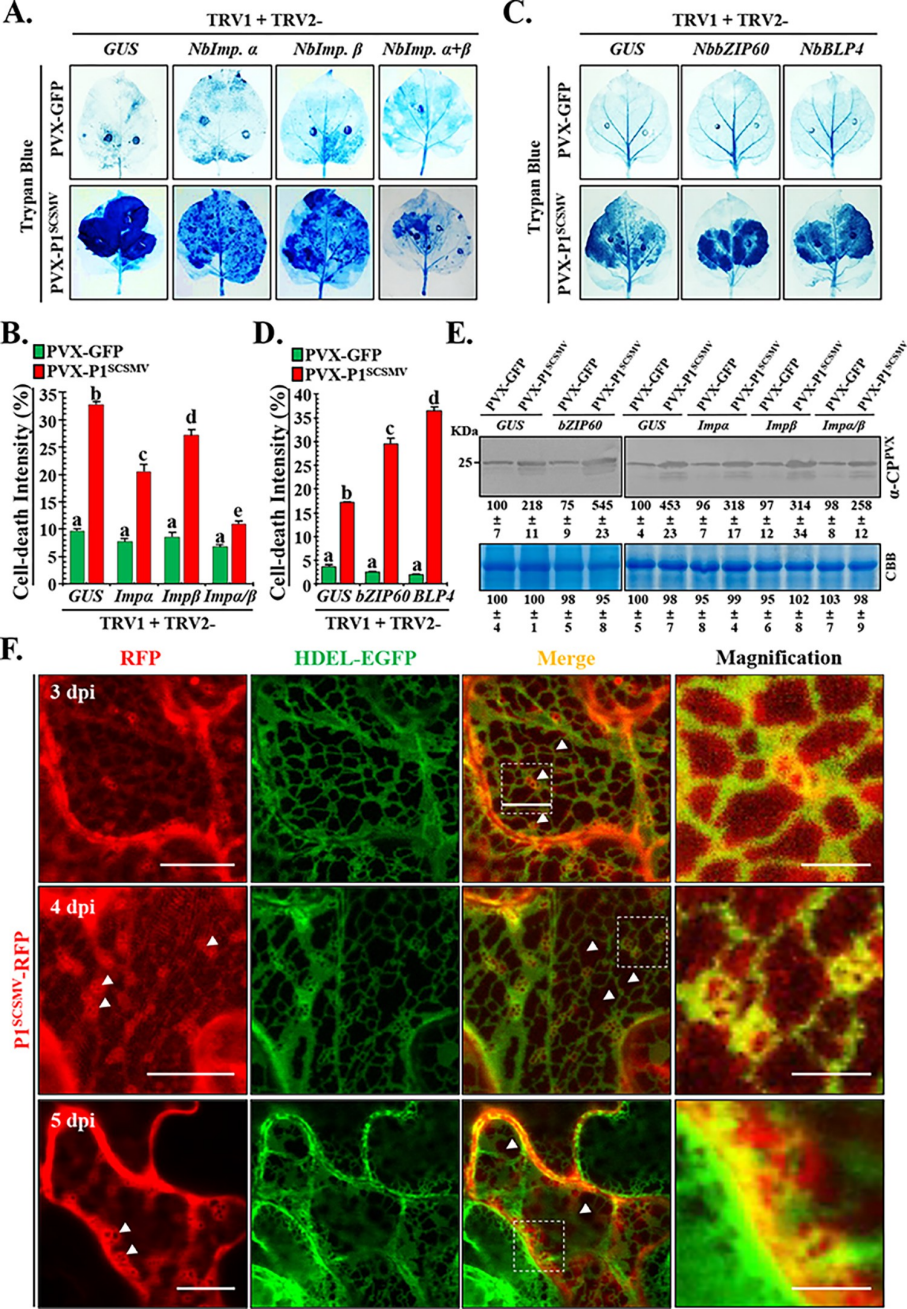
Analysis of the relationships between cell-death intensity, and nucleocytoplasmic-shuttling of P1^SCSMV^, IRE1/bZIP60 UPR signaling pathway, and morphological changes of ER. **(A).** The cell-death intensity of the PVX-GFP and PVX-P1^SCSMV^ in leaves that the nuclear importin system was partially inhibited. After 12 dpi of TRV inoculated, PVX-GFP and PVX-P1^SCSMV^ infiltration was performed, and the leaves were collected for trypan blue staining at the following 6 dpi. **(B).** The cell-death intensity was quantified using the ImageJ software. Different letters (a, b, and c, et. al.) mean significant difference between these columns as described above. **(C).** The cell-death intensity of the PVX-GFP and PVX-P1^SCSMV^ infected *NbbZIP60-* and *NbBLP4-*downregulated *N*. *benthamiana* plants **(D).** Quantification of the cell-death. Different letters (a, b, and c, et. al.) represent the same meaning above. **(E).** Accumulation levels of the PVX-GFP and PVX-P1^SCSMV^ in *N*. *benthamiana* plants that with and without *NbImp*. *α-*, *NbImp*. *β*, and *NbImp*. *α+β*, and *NbbZIP60-*downregulated condition were determined. The intensity of bands was quantified. The experiment was repeated for independent triple times, and the quantification was performed from independently three images at least. **(F)** Subcellular localization of the P1^SCSMV^ via overexpressing on the 16C transgenic *N*. *benthamiana* at 3 dpi, 4 dpi, and 5 dpi. The RFP monitor the P1^SCSMV^, and the GFP-HDEL was used to show the ER morphology. Bar scale, 10 μm. White arrow indicates the positions described in the result. White frame was enlarged in last lane (bar scale, 1 μm).

Previous studies have demonstrated the contribution of nucleocytoplasmic shuttling of P1^SCSMV^ to cell death in PVX-infected plants. However, the NLS peptides of P1^SCSMV^ determine the nuclear import dependence on the importin α/β transport system in plants. To clarify the relationship between the cell death phenotype caused by P1^SCSMV^ and the IRE1-bZIP60-associated UPR pathway in *N*. *benthamiana*, we inhibited the nuclear import of the P1^SCSMV^ through TRV-based silencing of *importin α/β* genes, and then inoculated the PVX-GFP and PVX-P1^SCSMV^ after genome-wide alignment to *N*. *benthamiana* (http://solgenomics.net/tools/blast/index.pl) using the importin α (KJ808745.1) and importin β (XM016585241.1) from *N*. *tabacum*. We obtained eight sequences (**S3A Fig**). These sequences were classified as Nbimp. α-1 (2 sequences), Nbimp. α-2 (2 sequences), Nbimp. β-1 (2 sequences), and Nbimp. β-2 (2 sequences) (**S3A Fig**). The homologous sequences of *Nbimp*. *α-1* and *Nbimp*. *α-2* were selected as targets for TRV-based silencing (TRV:*Nbimp*.*α*), and a similar procedure was followed for the *Nbimp*. *β* gene (TRV:*Nbimp*. *β*) (**S1 Table**). The TRV: *Nbimp*.*α+β* indicates the treatments of equal volumes of *Agrobacterium* suspension mixtures of TRV:*Nbimp*.*α* and TRV:*Nbimp*. *β*. At 12 dpi, except for symptoms caused by TRV infection, the *NbPDS*-silenced plants exhibited photo-bleaching, and the *importin α/β*-knockdown plants showed no visible abnormalities (**S3B Fig**). Semi-qPCR was performed to determine the relative expression levels of the target genes in the upper leaves (**S3C Fig**). Accordingly, *importin α*, *importin β*, and *importin α/β* were knocked down in these TRV-infiltrated plants. These target gene-silenced leaves were also inoculated with PVX-GFP and PVX-P1^SCSMV^. At 6 dpi, intensity of cell-death in the leaves was measured using trypan blue staining (**Fig 8A**). The TRV:*GUS*-infiltrated plants served as mock control, and the TRV:*Nbimp*.*α*-, TRV:*Nbimp*.*β*-, TRV:*Nbimp*.*α+β-*infiltrated plants were treated as described in the experiments (**Fig 8A**). Areas on the leaves were quantified using ImageJ software, and the cell-death intensity was calculated accordingly (**Fig 8B**). Cell-death intensity of all PVX-P1^SCSMV^-inoculated plants was found to be more severe than that of PVX-GFP-infiltrated plants. Cell death intensity was also alleviated in the PVX-P1^SCSMV^-inoculated *Nbimp*.*α*-, *Nbimp*.*β*-, and *Nbimp*.*α+β-*knockdown plants compared with the *GUS*-silenced plants (**Fig 8A and 8B**). We also found that PVX-GFP and PVX-P1^SCSMV^-infected plants showed decreased virus accumulation levels in the *Nbimp*.*α*-, *Nbimp*.*β*-, and *Nbimp*.*α+β-*knockdown plants compared with the *GUS*-silenced plants (**Fig 8E, left**). In summary, these results indicate that the NLS peptide-mediated nuclear import of P1^SCSMV^ is required for virus accumulation and cell death in *N*. *benthamiana*.

Next, the expression levels of *NbbZIP60* and *NbBLP4*, marker genes for the IRE1-bZIP60-associated UPR signaling pathway were determined to identify whether the cell-death phenotype was caused by IRE1-bZIP60-associated UPR signaling inhibition in *N*. *benthamiana* (**S4A Fig**). When the *NbPDS*-silenced plants exhibited photobleaching phenotype at 10 dpi, we performed semi-qPCR to determine the relative expression levels of *NbbZIP60* and *NbBLP4* genes in the upper leaves of the TRV-infiltrated plants. As expected, the target genes were found to be downregulated (**S4B Fig**). We then inoculated PVX-GFP and PVX-P1^SCSMV^ into target gene-silenced leaves. At 6 dpi, the leaves were collected and subjected to trypan blue staining, and the cell-death area was calculated using the ImageJ software (**Fig 8C and 8D**). Cell death intensities in all PVX-P1^SCSMV^-inoculated plants were found to be more severe than that in the PVX-GFP-inoculated plants. Cell death intensity in the *NbbZIP60* and *NbBLP4*-silenced plants was also more severe than that in the *GUS*-knockdown plants under PVX-P1^SCSMV^ infection (**Fig 8C and 8D**). Western blotting was performed to determine the PVX accumulation using anti-CP^PVX^ specific antibodies. PVX-GFP accumulation levels in *NbbZIP60*-silenced plants were found to be higher than those in *GUS*-silenced plants (**Fig 8E, right**), indicating that the UPR signaling pathway contributes to PVX accumulation. Under PVX-P1^SCSMV^ infection conditions, *NbbZIP60*-silenced plants showed higher virus accumulation than *GUS*-knockdown plants (**Fig 8E**), indicating that P1^SCSMV^ enhanced PVX accumulation in a manner dependent on IRE1-bZIP60-associated UPR signal transduction.

To identify the role of P1^SCSMV^ in cell death during PVX infection, we further explored the finer details of subcellular localization of P1^SCSMV^ by overexpressing RFP-tagged P1^SCSMV^ at the C-terminus on the epidermal leaves of 16C transgenic *N*. *benthamiana* (**Fig 8F**). The EGFP protein of 16C transgenic plants contains a conventional ER signal peptide (HDEL) that targets ER. These findings showed that free RFP protein did not co-localize with the polygonal meshes of the ER (**S1B Fig**), whereas P1^SCSMV^-RFP impeccably merged with the polygonal meshes of the ER, and formed small vesicles at the joint of the polygonal meshes at 3 dpi (**Fig 8F, upper**). At 4 dpi, the co-localization of P1^SCSMV^-RFP with the polygonal meshes was partially disrupted, and the number of vesicles was increased in the ER polygonal meshes (**Fig 8F, middle**). At 5 dpi, the co-localization disappeared completely, and the ER polygonal meshes were distorted and collapsed (**Fig 8F, bottom, white arrows**). These results demonstrate that P1^SCSMV^ was localized to both the nucleus, cytoplasm, and polygonal meshes of the ER. At 5 dpi, these small vesicles associated with and moved to the cell membrane (**Fig 8F, white arrows, RFP channel**), and the polygonal meshes collapsed to form large black holes in the ER (**Fig 8F, white arrows, merge channel**). These results strongly suggest that P1^SCSMV^ directly induces the distortion and collapse of ER polygonal meshes, and ultimately lead to cell-death.

### NLS of P1^SCSMV^ and the stem-loop splicing region of *bZIP60U* are crucial for P1^SCSMV^ inhibition of *bZIP60U* mRNA splicing

**Fig 9:**
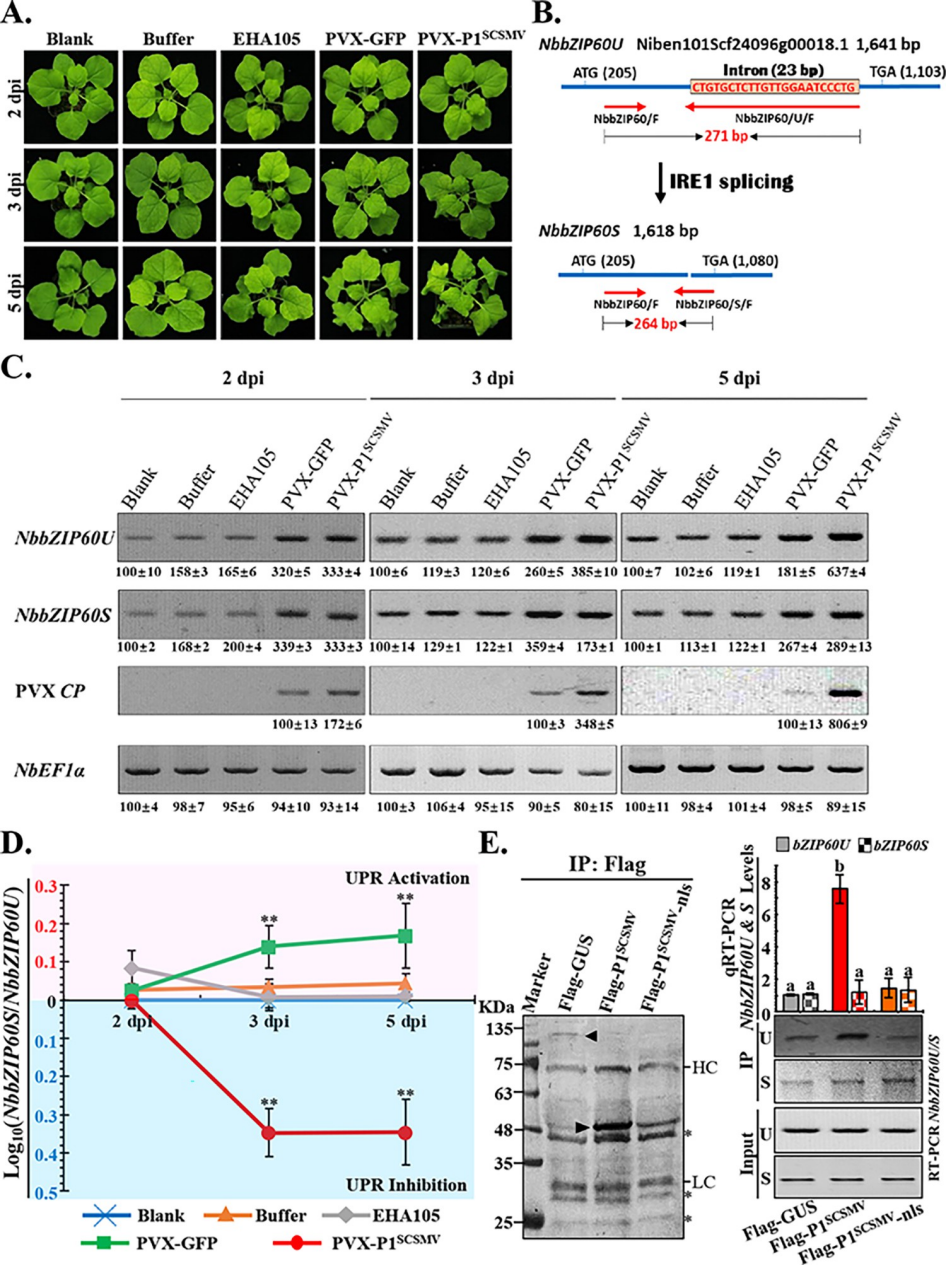
P1^SCSMV^ inhibited the IRE1-mediated splicing of the *NbbZIP60U* through directly binding to the stem-loop of *NbbZIP60U in vivo*. **(A).** Phenotype of the *N*. *benthamiana* plants with different treatments at 2, 3, and 5 dpi. **(B).** Illustration of IRE1-mediated splicing of *NbbZIP60U* (Niben101Scf24096g00018.1) to *NbbZIP60S*. The start and end of each type of *NbbZIP60* were marked and the red arrows represent the pairs of primers designed for the analyses. Red number indicate the lengths of the amplified fragments with the target primers. **(C).** Quantification of the relative levels of *NbbZIP60U* and *NbbZIP60S* in the five-treated *N*. *benthamiana*. The levels of PVX *CP* represents the virus accumulation levels. The experiment was independently repeated for three times, and quantification was performed using at least three independent images. **(D).** The activation and inhibition of the IRE1/bZIP60 UPR signaling pathway were calculated based on logarithm value of *NbbZIP60U*/*NbbZIP60S* in base-10. The upper pink panel represents the UPR activation, and the lower cyan panel indicates UPR inhibition. ** means significant difference with a *p*-value < 0.01. **(E).** An RNA-immuno-precipitation assay (RIP) showed the binding of P1^SCSMV^ to the stem-loop of *NbbZIP60U* through its NLS region *in vivo*. HC and LC denote “heavy chain” and “Light chain”, respectively. The right panel shows the relative levels of *NbbZIP60U* and *NbbZIP60S* using RT-PCR and qRT-PCR analyses of the input and precipitated (IP) samples. In the RT-PCR analyses of IP samples, the PCR cycle was set to 45 for the amplification of the *NbbZIP60S* and 25 cycles for *NbbZIP60U*. In the qRT-PCR analyses, the column filled with solid colors represents the amount of *bZIP60U* isolated from precipitated Flag-GUS, Flag-P1^SCSMV^, and Flag-P1^SCSMV^-nls, while the column filled with mosaic colors indicates the amount of *bZIP60S* isolated from precipitated proteins. Letters (a and b) indicate significant differences between the columns.

To explore the mechanism by which P1^SCSMV^ inhibits the IRE1-bZIP60-associated UPR signaling pathway, we transferred PVX-GFP and PVX-P1^SCSMV^ into *N*. *benthamiana* through *Agrobacterium*-mediated infiltration. Healthy, *Agrobacterium* suspension buffer-infiltrated plants, and blank *Agrobacterium* EHA105-infiltrated plants served as controls (**Fig 9A**). The plants were photographed at 2 dpi, 3 dpi, and 5 dpi (**Fig 9A**). Disease in the PVX-GFP and PVX-P1^SCSMV^-infiltrated plants was observed to become increasingly severe (**Fig 9A**). Based on the reported splicing sites of the *NbbZIP60U*, we designed two pairs of primers: NbbZIP60/F and NbbZIP60/U/R, and NbbZIP60/F and NbbZIP60/S/F. The amplified 271 bp and 264 bp fragments represented *NbbZIP60U* and *NbbZIP60S* mRNA, respectively (**Fig 9B**). We also determined the expression levels of the *NbbZIP60U*, *NbbZIP60S*, and PVX *CP* in the leaves with infiltration mentioned above by RT-PCR at 2, 3, and 5 dpi. The *NbEF1α* gene was used as internal control (**Fig 9C**). More *NbbZIP60* mRNA accumulated in PVX-P1^SCSMV^-infected plants than in PVX-GFP-infected plants at 3 and 5 dpi (**Fig 9C**). The ImageJ software was used to quantify the intensity of the RT-PCR bands on the gels. We calculated the expression ratio of *NbbZIP60U* to *NbbZIP60S*, and the value of the logarithm (base ten) was obtained (**Fig 9D**). We split Fig 9D into two parts: the area above zero (pink panel), indicates IRE1-bZIP60-associated UPR signaling activation, whereas that below zero (cyan panel), represents the suppression of IRE1-bZIP60-associated UPR signaling. PVX-P1^SCSMV^-infected plants were found to display severe inhibition of the IRE1-bZIP60-associated UPR signaling pathway at 3 and 5 dpi, whereas the IRE1-bZIP60-associated UPR signaling pathway was activated in PVX-GFP-infected plants from 3 to 5 dpi (**Fig 9D**). These results demonstrate that P1^SCSMV^ inhibits the splicing of *NbbZIP60U* to *NbbZIP60S* mRNAs.

Previous studies have shown that P1 of wheat streak mosaic virus (P1^WSMV^) binds to dsRNA in a non-specific manner [40,52]. WSMV and SCSMV belong to the same genus (*Poacevirus*), and encode highly similar P1 proteins [53]. Both P1^SCSMV^ and P1^WSMV^ act as VSRs that function facilitate the defense against the antiviral immunity of host plants [36]. Hence, we speculated that P1^SCSMV^ directly binds to *NbZIP60U* mRNA in the presence of PVX infection, and inhibits the splicing of *NbZIP60U* mRNA. To test this hypothesis, we performed RNA immunoprecipitation (RIP) using commercial flag beads in the presence of PVX-P1^SCSMV^ and PVX-P1^SCSMV^-nls infection (**Fig 9E**). The results showed that Flag-tagged GUS, P1^SCSMV^, and P1^SCSMV^-nls proteins were successfully expressed and precipitated (**Fig 9E, left, black arrowhead**). Furthermore, we purified the RNAs bound to these precipitated proteins. RT-PCR and qRT-PCR were performed to determine the relative expression levels of the target genes using specific pairs of primers. The results showed that *NbZIP60U* mRNA was enriched almost 10-fold by P1^SCSMV^ compared to that by GUS and P1^SCSMV^-nls (**Fig 9E, right, upper**). In addition, *NbZIP60S* mRNA bound to these Flag-tagged proteins could only be barely detected following 45 cycles in the RT-PCR assays (**Fig 9E, right, bottom**). These data suggest that the NLS peptide on P1^SCSMV^ and the stem-loop splicing region of *NbZIP60U* mRNA are crucial for P1^SCSMV^-mediated *NbZIP60U* mRNA splicing inhibition.

**Fig 10:**
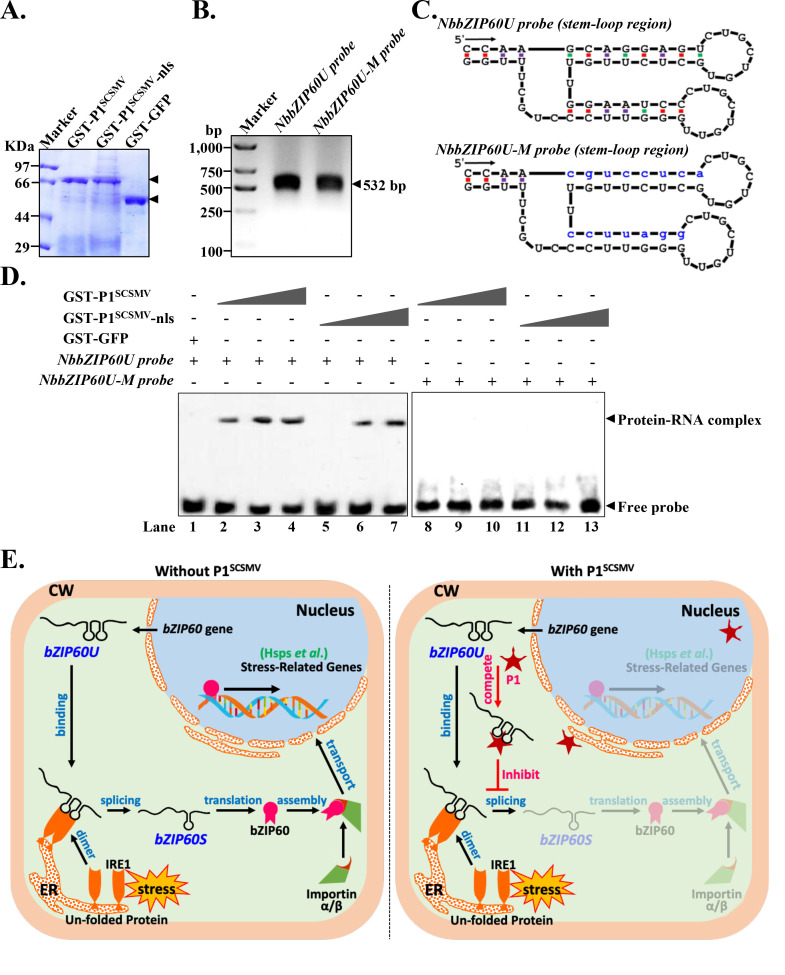
P1^SCSMV^ directly binds to the stem-loop region of *NbbZIP60U* through via NLS region *in vitro*. **(A).** CBB staining of the purified recombinant GST-tagged GFP, P1^SCSMV^, and P1^SCSMV^-nls mutant. Black arrows indicate the target protein. **(B).** *In vitro* transcription of the 532-bp 5’-biotinylated stem-loop region containing RNA from *NbbZIP60U* (*NbbZIP60U probe*) and its corresponding mutant (*NbbZIP60U-M probe*). **(C).** Predicted stem-loop regions located in *NbbZIP60U* transcripts and their mutants. Blue letters in *NbbZIP60U-M probe* represent the mutations. **(D).** *In vitro* electrophoretic mobility shifts assay was used to analyses the direct binding of GST-P1^SCSMV^ to the stem-loop containing single-stranded RNA by the NLS peptide. The black triangle indicates the gradually increased concentration of the target protein, and the protein concentration gradient of the GST-P1^SCSMV^ and GST-P1^SCSMV^-nls used were the same (lane 2 & 5, 0.1 μg, lane 3 & 6, 0.5 μg, and lane 4 & 7, 2.5 μg). The labeled probes (*NbbZIP60U probe* & *NbbZIP60U-M probe*) used were 1 μL in all lanes. The plus sign represents the lane containing the target protein and corresponding probe. **(E)**. The proposed working model of the P1^SCSMV^ in achieving moderate robust infection. In absence of P1^SCSMV^, UPR activation leads to increased expression of *bZIP60U*. *bZIP60U* then further processed by the ER-localized IRE1 protein to form *bZIP60S*. Normal translation generated bZIP60S transcription factor will import to the nuclear by the cellular importin α/β system, then mediated expression of stress-related genes and promoted pro-survival of the cell. In UPR activation with P1^SCSMV^ condition, the *bZIP60U* induced by UPR activation directly bound by the P1^SCSMV^. The optimal and correct splicing of *bZIP60U* was inhibited, leading to decreased *bZIP60S* and bZIP60S protein. All of these decreased the intensity of the bZIP60S-mediated stress-related genes expression, and finally caused PCD of the cell.

To further demonstrate the direct binding of P1^SCSMV^ to *NbZIP60U* mRNA *in vitro*, we performed an electrophoretic mobility shift assay (EMSA) as well. First, we purified GST-tagged GFP, P1^SCSMV^-nls, and P1^SCSMV^ from *E*. *coli* BL21 (**Fig 10A**). Then, based on the predicted secondary RNA structures of the *NbbZIP60U* transcript (**S3 Table**) [17], we identified the base-pair of the two predicted stem-loop regions of *NbbZIP60U*, and mutated the bases to generate the *NbbZIP60U-M* transcript for disrupting the base-pair of the stem-loop (**Fig 10C**). The 532-bp length partial *NbbZIP60U* fragment containing the stem-loop splicing region and its mutant *NbbZIP60U-M* were transcribed and labelled with digoxin *in vitro* (**Fig 10B**). The EMSA results showed that GST-P1^SCSMV^ bound more *NbbZIP60U probe* than GST-P1^SCSMV^-nls at the same protein concentrations (**Fig 10D, lane 2 to 5, 3 to 6, and 4 to 7**), while neither GST-P1^SCSMV^ nor GST-P1^SCSMV^-nls were bound to the *NbbZIP60U-M probe* (**Fig 10D, lane 8 to 11, 9 to 12, and 10 to 13**). Furthermore, we transiently expressed Flag-GUS and Flag-P1^SCSMV^ in the UPR inducer DTT and tunicamycin application, and examined the processing of internal *NbbZIP60U in vivo* using self-prepared polyclonal anti-NbbZIP60 antibodies (**S6 Fig**). The results showed that P1^SCSMV^ specifically inhibits the proteolytic processing of *NbbZIP60U* with and without the application of the UPR inducer. In summary, our results demonstrated that P1^SCSMV^ binds to the stem-loop splicing region of *NbbZIP60U* with its NLS peptide, and inhibits IRE1-mediated *NbbZIP60U* splicing and IRE1-associated UPR signaling pathways.

## Discussions

Most viral proteins are synthesized on the ER surface by host ribosomes. This generates large amounts of unfolded/misfolded proteins in the ER lumen, and thus leading to ER stress. ER stress activates a variety of cellular protective responses, including the UPR signaling pathway. The UPR signalling pathway promotes cell survival by alliviating ER stress through synthesis of chaperones aid peotein folding. However, UPR signaling pathway can also promote programmed cell death when overwhelmed by ER stress. Previous studies showed that several viral protein activate the UPR and promote the *bZIP60* expression, such as pTGB2^PVX^, pTGB3^PVX^, 6K2^TuMV^, 6K2 of potexviruses, βC1^TYLCCNV/TYLCCNB^, P10^RBSDV^, and P11^GVX^ [15,17,22–25,54]. In addition, Li et al. (2021) reported that rice stripe virus infection elicits the UPR pathway. In response to this, the movement protein, NSvc4, can hijack the UPR-activated type-I J-domain protein (NbMIP1s), to protect itself from degradation via the host UPR-activated autophagy pathway [55]. Here, we found that the P1^SCSMV^ acts as a classical VSR that suppresses both local and systemic RNA silencing (**Fig 1**). The P1 protein encoded by the genus *Poacevirus* has been reported to act as a VSR that can suppress local RNA silencing [36,37]. Our results revealed a similar systemic RNA silencing suppression ability of P1^SCSMV^. Furthermore, we confirmed that heterogeneous expression of P1^SCSMV^ by PVX promoted programmed cell death (PCD) and enhanced recombinat virus accumulation compared to the PVX-GFP infection in *N*. *benthamiana* (**Fig 2**), which provided further details on the previously proposed disease-enhancing role of poaceviruses’ P1 by *cis-*heterogeneous-mediated expression via PVX [38,39]. These results demonstrate that P1^SCSMV^ acts as both a VSR to determine the fate of viral infection and disease symptoms regulator in plants. Therefore, we also investigated the mechanisms by which P1^SCSMV^ triggered PCD during PVX infection in *N*. *benthamiana*.

For this purpose, we first quantified the relative expression levels of IRE1/bZIP60 UPR signalling pathway-related marker genes in the presence of P1^SCSMV^ under UPR activation (**Fig 3D**) and resting conditions (**Fig 3E**). The results showed that PVX-GFP infection promoted the expression of UPR marker genes [15], which is supported by previous studies showing activating the UPR signalling pathway by pTGB2^PVX^ and pTGB3^PVX^ [23,24]. We also found that the expression of UPR marker genes in PVX-P1^SCSMV^ infection was significantly different from that in PVX-GFP infection (**Fig 3D, green column**), indicating that the pTGB2^PVX^- and pTGB3^PVX^- activated UPR signalling pathway was suppressed by P1^SCSMV^. Furthermore, we transiently overexpressed the P1^SCSMV^ under resting UPR conditions (**Fig 3E**). We found that compared to the mock-treatment and GFP-expression conditions, P1^SCSMV^ slightly suppressed the expression of the downstream marker genes *CAM* and *BLP4* (**Fig 3E, red column**). These results demonstrated that P1^SCSMV^ may play a suppressive role in the IRE1/bZIP60 UPR signaling pathway during recombinant PVX-P1^SCSMV^ infection.

To explore the mechanism used by P1^SCSMV^ to suppress the IRE1/bZIP60 UPR signaling pathway, we first investigated the subcellular localization of P1^SCSMV^ (**Fig 3E**). The subcellular localization of P1^SCSMV^ was identical to that of the free RFP, which was found in both the cytoplasm and nucleus (**Fig 3E**). This facilitate the consideration regarding which localization type is essential for the IRE1/bZIP60 UPR signaling pathway suppression roles for P1^SCSMV^. Many plant viruses encoding VSRs, such as βC1^TYLCCNV/TYLCCNB^ [12,54], γb^BSMV^ [5,56], 2b^CMV^ [57], and NS3^RSV^ [10,58], can localize to both the cytoplasm and nucleus. Any changes in subcellular localization will reduce VSR actvity. In addition, the localization of 2b^CMV^ [57], NS3^RSV^ [58], and βC1^TYLCCNV/TYLCCNB^ [12,54,59] to the nucleus often depends on the NLS and host nuclear importin system. Hence, we investigated whether the P1^SCSMV^ also had similar characteristics. Through bioinformatics prediction and mutation analyses, we confirmed that P1^SCSMV^ had a bipartite NLS between aa position of 251 to 254 (NLS1), and 257 to 263 (NLS2) (**Fig 4**). Furthermore, we also found that P1^SCSMV^ could interact with itself *in vitro* and *in vivo* (**Fig 5**), similar to the VSRs mentioned above [6,10,13]. Mutations of NLS1, NLS2, or NLS also resulted in direct disruption of the self-interactions (**Fig 5D**), leading to complete loss of VSR activity (**Fig 6**). Compulsion of the nuclear importin or exportin of P1^SCSMV^ by adding the canonical NLS (“PKKKRKV”) or nuclear exportin signal (NES, “ELALKLAGLDIN”) at the N-terminus leads to complete loss of its VSR activity (**Fig 6**). These results demonstrate that NLS is essential for the self-interaction and VSR activty of P1^SCSMV^, and both the cytoplasmic and the nuclear localization (cytoplasmic-nuclear shuttling) are important for its VSR activity, similar to the findings on the 2b^CMV^ [57] and βC1^TYLCCNV/TYLCCNB^ [54,59].

Previous studies have showwn that P1^SCSMV^ plays a disease-enhancing role when expressed by recombinant PVX-P1^SCSMV^ in *N*. *benthamiana* [39]. To further explore the effect of NLS mutations on the disease-enhancing role of P1^SCSMV^, we *cis-*heterologously expressed P1^SCSMV^ and its NLS mutant derivatives using recombinant PVX in *N*. *benthamiana*. Hydrogen peroxide accumulation, PCD intensity, and virus accumulation were determined by DAB staining, trypan blue staining, and tissue printing, respectively (**Fig 7A**). Furthermore, virus accumulation levels and marker genes of the UPR signalling pathway were evaluated by northern blotting, western blotting, and qRT-PCR in the same systemic leaf (**Fig 7B–7F**). All these results demonstrated that NLS1 of P1^SCSMV^ is essential for hydrogen peroxide accumulation, PCD, and recombinant virus accumulation (**Fig 7B–7E**), whereas NLS2 is only significant for virus accumulation (**Fig 7D and 7E**). These obseved physiological indices of different P1^SCSMV^ mutants are highly consistent with the VSR activities shown in Fig 6, and all conclusions are also in line with other viral VSRs that have an NLS, such as 2b^CMV^ [57], and βC1^TYLCCNV/TYLCCNB^ [59], and NS3^RSV^ [10]. We further performed qRT-PCR to determine the relative expression levels of UPR signalling pathway marker genes (**Fig 7F**). The results showed that in the presence of PVX infection, the different P1^SCSMV^ NLS mutants appeared to promote the expression of marker genes compared to the mock-treated group, whereas PVX-P1^SCSMV^ decreased the expression of UPR marker genes (**Fig 7F**). These results once again suggest that PVX infection activated the IRE1/bZIP60 UPR signalling pathway via its pTGB2 and pTGB3, as described previously [23,24], and demonstrate that the NLS of wild-type P1^SCSMV^ plays a significant role in the suppression of the IRE1/bZIP60 UPR signaling pathway.

Hundreds of cellular proteins with a clasisical NLS that translocate to the nuclear depend on the importin α/β heterodimer, and the importin α often directly binds to the classical NLS on the carge proteins [60]. Hence, we wanted to knock down the *importin α/β* in *N*. *benthamiana* by tobacco rattle virus-mediated gene silenicng [61,62] and to observe the PCD intensity caused by recombinant PVX-P1^SCSMV^ infection (**Fig 8A, 8B, and 8E**). We found that the nuclear translocation of P1^SCSMV^ by knocking down the *NbImp*. *α*, *NbImp*. *β*, or both *NbImp*. *α* and *NbImp*. *β*, were alleviated the PCD intensity (**Fig 8A, 8B, and 8E**). These results, together with the results in Fig 7, strongly suggest that the NLS or nuclear translocation of P1^SCSMV^ is required for PCD, which implies that PCD is tightly associated with the NLS of P1^SCSMV^.

To confirm whether PCD was caused by the inhibition of the IRE1/bZIP6 UPR signalling pathway, we further explored the subcellular localization of P1^SCSMV^ and the relationship between PCD and the IRE1/bZIP6 UPR signalling pathway by knocking down the marker genes *NbbZIP60* and *NbBLP4* (**Fig 8C–8E**). When the IRE1/bZIP6 UPR signalling pathway was inhibited by knocking dwon *NbbZIP60* and *NbBLP4*, PCD caused by the recombinant virus PVX-P1^SCSMV^ infection was more severe (**Fig 8C and 8D**), and virus accumulation levels were also increased (**Fig 8E**). These results were completely contrary to those of most plant viruses’ infections that could activate the UPR signaling pathway, such as TYLCCNV/TYLCCNB [54], TuMV [17], and GVX [22], in which silencing or knocking out *NbbZIP60* expression decreased virus accumulation. Our results were in line with those of PVX-GFP and PVY-GFP infection when *NbbZIP60* and *BI-1* expression were knocked down in *N*. *benthamiana* [22]. These results strongly demonstrate that P1^SCSMV^-induced PCD is associated with IRE1/bZIP6 UPR signalling pathway activation. Proteins that trigger the UPR often reside on the ER [15,17]. Therefore, we examined the subcellular localization of the P1^SCSMV^ by *Agrobacterium*-mediated expression of P1^SCSMV^-RFP. We observed that P1^SCSMV^ can also localise to the ER. P1 was uniforml localized to the ER polygonal meshes at 3 dpi, which then formed vesicles and moved to the joint of the polygonal meshes at 4 dpi. At 5 dpi, P1^SCSMV^ formed small vesicles associated with the cell membrane, and the ER polygonal meshes collapsed, forming numerous large black holes (**Fig 8F**). These observations suggest that the PCD caused by P1^SCSMV^ was determined by ER localization-triggered pathological changes, which also indicated that the PCD caused by P1^SCSMV^ was associated with IRE1/bZIP6 UPR signalling pathway activation.

The synthesis of NbbZIP60, an active transcription factor, depends on the optimal and correct splicing of *NbbZIP60U* and formation of *NbbZIP60S* in the ER [17]. The NbbZIP60 protein translated from *NbbZIP60S* enterss the nucleus, and regulates the expression of various downstream genes associated with the UPR signaling pathway [63]. We hypothesized that P1^SCSMV^ directly binds to *NbbZIP60U* during UPR activation, inhibits *NbbZIP60U* splicing, and decreases NbbZIP60 protein expression levels, to ultimately block the cell-survival signal from the ER to the nucleus. To test this hypothesis, we first inoculated the PVX-P1^SCSMV^ and PVX-GFP into the *N*. *benthamiana* by *Agrobacterium*-meidated infiltration (**Fig 9A**), followed by RT-PCR and RIP-associated qRT-PCR analyses at an early stage (**Fig 9C**). Specific pairs of primers used for RT-PCR and qRT-PCR were designed based on the splicing sites (**Fig 9B**). RT-PCR results showed that PVX-P1^SCSMV^ and PVX-GFP infection activated the UPR signaling pathway, as evidenced by significantly increased *NbbZIP60U* accumulation compared to those observed in other control groups. Less *NbbZIP60S* was found to accumulate under PVX-P1^SCSMV^-infected conditions than under PVX-GFP infection at 3 dpi (**Fig 9C, middle**). Furthermore, we performed RT-PCR analyses in three indepentdent replicates. The *NbEF1α* was treated as an internal control, and bands on RT-PCR images were quantified by ImageJ software. The relative ratios of *NbbZIP60S/NbbZIP60U* were calculated, and values were reported in base-10 logarithm, as shown in Fig 9D. Values above zero (pink panel) indicate UPR activation, and those below zero represent UPR inhibition. The results indicated that PVX-GFP infection significantly activated the UPR signalling pathway at 3 and 5 dpi (**Fig 9D**), which has also been observed in previous studies [22–24]. Under conditions of PVX-P1^SCSMV^ infection, the activated UPR signaling pathway was dramatically suppressed at 3 and 5 dpi, as shown in Figs 7F and 8. RIP-qRT-PCR results demonstrated that P1^SCSMV^ was capable of binding to the *NbbZIP60U in vivo*, yet barely bound to *NbbZIP60S* (**Fig 9E**). In addition, we found that the *NbbZIP60U* binding ability of P1^SCSMV^ depended on its NLS (**Fig 9E**). Furthermore, we purified the recombinant GST-tagged P1^SCSMV^ and its derivatives, and prepared a biotin-labelled 532-bp length *NbbZIP60U* partial fragment that contained the stem-loop splicing sites and its mutant *NbbZIP60U-M* via *in vitro* transcription, as previously described for dsRNA helicase unwinding experiments [5] (**Fig 10A and 10B**). EMSA results suggested that GST-tagged P1^SCSMV^ could bind to the biotin-labelled 532-bp *NbbZIP60U probe* in a dose-dependent manner, depending on its NLS *in vitro* (**Fig 10C, left panel**), whereas both GST-P1^SCSMV^ and GST-P1^SCSMV^-nls could not bind to the *NbbZIP60U-M probe* (**Fig 10C, right panel**). Furthermore, we also observed the processing of the internal *NbbZIP60U in vivo* as reported previously [64], which showed transient expression of the Flag-P1^SCSMV^ upon UPR activation (**S6 Fig**). These results show that P1^SCSMV^ inhibits the splicing of *NbbZIP60U in vivo*, and affects the amount of spliced *bZIP60*.

Based on the results obtained in the present study and previous reserch, we propose a mechanism by which P1^SCSMV^ inhibits the UPR and triggers PCD under conditions of UPR activation (**Fig 10E**). Without P1^SCSMV^, UPR activation leads to an increased expression of *bZIP60U*. Then, the *bZIP60U* is processed by the ER-localized IRE1 protein to form *bZIP60S*. The generated bZIP60S transcription factor is imported into the nucleusnucleus by the cellular nuclear importin α/β system, mediating the expression of stress-related genes and promoting the survival of the cell (**Fig 10E, left**). In the presence of P1^SCSMV^, *bZIP60U* induced by UPR activation drectly binds to P1^SCSMV^. Optimal and correct splicing of *bZIP60U* is inhibited, which subsequently leads to decreased *bZIP60S* and bZIP60S protein levels. Thus, the intensity of bZIP60S-mediated stress-related gene expression is decreased, and ultimately causing PCD in the cell (**Fig 10E, right**). During SCSMV infection in sugarcane, the 6K2 protein likely activates the UPR signalling pathway [17], which encodes molecular chaperones that are beneficial for protein folding efficiency and SCSMV virus accumulation [26,54]. However, in long-term arm races and evolution, SCSMV-encoded P1, acts as a brake to prevent severe ER stress-induced PCD by directly inhibiting *bZIP60U* splicing. Our results demonstrate a distinct pathogenicity mechanism of a viral multifunctional protein through manipulation of the UPR pathway to fine-tune the functions of NbbZIP60, and thereby maintain moderate UPR activation to restrict uncontrolled potyvirus infection. Our results thus highlight the multifunctionality of virus-encoded VSRs, and may guide furture research efforts on potyvirus resistence and high-yield sugarcane cultivation base on inhibition of the bZIP60.

## Supporting information

S1 Fig Prediction of the NLS of P1^SCSMV^ and the subcellular localization of free RFP in *N*. *benthamiana*. **(A).** Prediction of the NLS of P1^SCSMV^ using the online NLS_Mapper server. **(B**). Subcellular localization of the free RFP in the 16C transgenic plant. Bar scale, 10 μm.
(JPG)

S2 Fig Expression of the P1^SCSMV^ and its NLS-related mutants in subcellular localization analyses. (TIF)

S3 Fig Phenotype, silencing efficiency, and virus accumulation levels analyses using the TRV-based gene silencing in condition of the PVX-GFP and PVX-P1^SCSMV^ infection. **(A).** Phylogenetic analysis all the encoding genes of importin α and importin β in*N*. *benthamiana*. Bar scale, 0.1. **(B).** Phenotype of silencing of *NbImp*. *α*, *NbImp*. *β*, and *NbImp*. *α + β* in *N*. *benthamiana* at 12 dpi. **(C).** The silencing efficiency were determined by the simi-quantitative PCR. The TRV-*gus*-inoculated plants were served as experimental control. All these PCRs were set to 30 cycles.
(JPG)

S4 Fig Phenotype and silencing efficiency analyses in condition of the PVX-GFP and PVX-P1^SCSMV^ infection. **(A).** Phenotype of silencing of *NbPDS*, *NbGUS*, *NbBLP4*, and *NbbZIP60* at 12 dpi. **(B).** The silencing efficiencies were determined by the simi-quantitative PCR. All PCRs runs set to 25 cycles.
(JPG)

S5 Fig Western blot detection of the P1^SCSMV^ and its mutants in yeast. The glyceraldehyde-3-phosphate dehydrogenase (GAPDH) was treated as loading control.
(TIF)

S6 Fig P1^SCSMV^ inhibit the proteolytic processing of *bZIP60U* response to ER stress. **(A).** P1^SCSMV^ inhibited *bZIP60U* processing to a great extent in the presence and absence of the UPR inducers (DTT and tunicamycin). The concentration of DTT, tunicamycin (Tm), and DMSO were 2 mM, 5 μg/mL, and 0.1%. DMSO was used as a solvent to dissolve DTT and tunicamycin. After two days (48 h) of infiltration, and DTT, Tm, and DMSO were spayed to the target leaves. Two-hour (2 h) later, the leaves were harvested and its total proteins were extracted. The OD_600_ of *Agrobacterium* expressing Flag-GUS and Flag-P1^SCSMV^ was set as 1.0. Polyclonal anti-NbbZIP60 and anti-FLAG antibodies were used to analyses proteins’ expression. **(B).** Gradient proteolytic processing of *bZIP60U* increased levels of P1^SCSMV^. *Agrobacterium* expressing Flag-P1^SCSMV^ with different OD_600_ values infiltrated the *N*. *benthamiana* leaves. The internal spliced and un-spliced forms of bZIP60 were qualified using self-prepared anti-NbbZIP60 antibodies.
(TIF)

S7 Fig Interactions analyses of the different P1 potential NLS mutants. Bar scale, 20 μm.
(TIF)

S8 Fig RNA silencing suppression activity assay of the different P1 potential NLS mutants. (TIF)

S1 Table Specific pairs of primers used in the study. (XLSX)

S2 Table Sequences of the RNA probes used in this study. (DOCX)

S3 Table Features of the *NbbZIP60U* mRNA sequences. (PDF)
