# Supplementary figures and images for: Sugarcane streak mosaic virus P1 protein inhibits unfolded protein response through direct suppression of bZIP60U splicing
Source: PLoS Pathog. 2023 Oct 26;19(10):e1011738. doi: 10.1371/journal.ppat.1011738 (PMC10697598; doi:10.1371/journal.ppat.1011738)

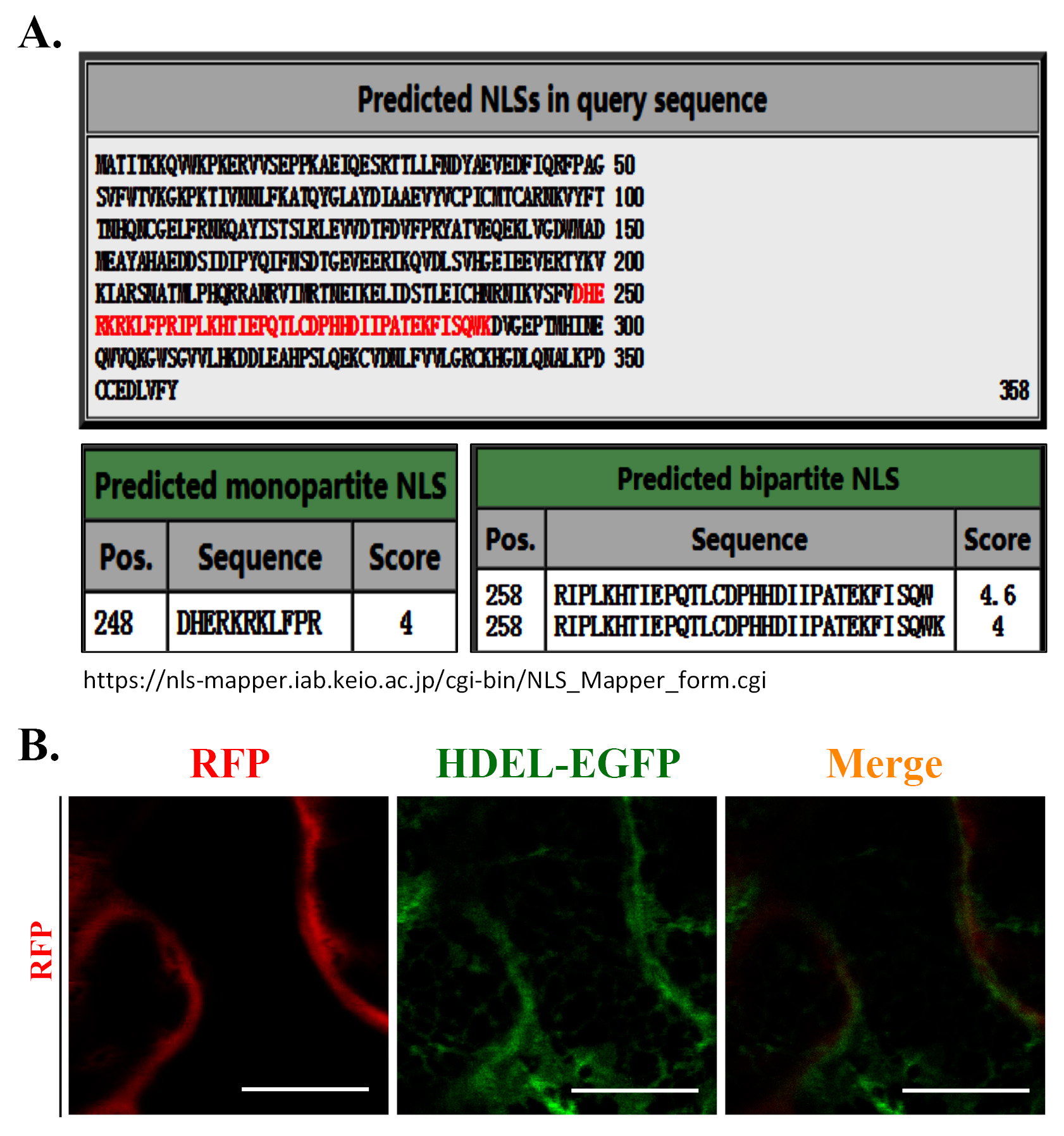

Supplement: S1 Fig — (A). Prediction of the NLS of P1SCSMV using the online NLS_Mapper server. (B). Subcellular localization of the free RFP in the 16C transgenic plant. Bar scale, 10 μm. (JPG) [file ppat.1011738.s001.jpg]

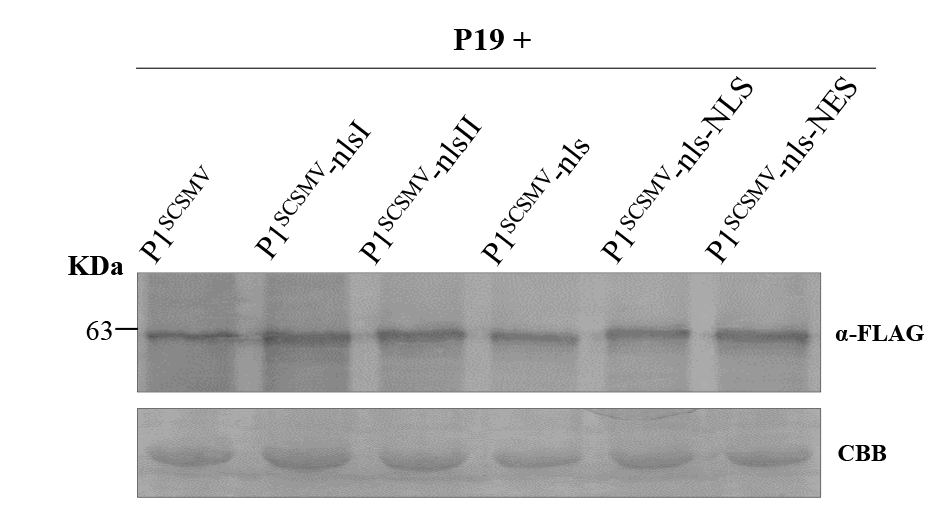

Supplement: S2 Fig — (TIF) [file ppat.1011738.s002.tif]

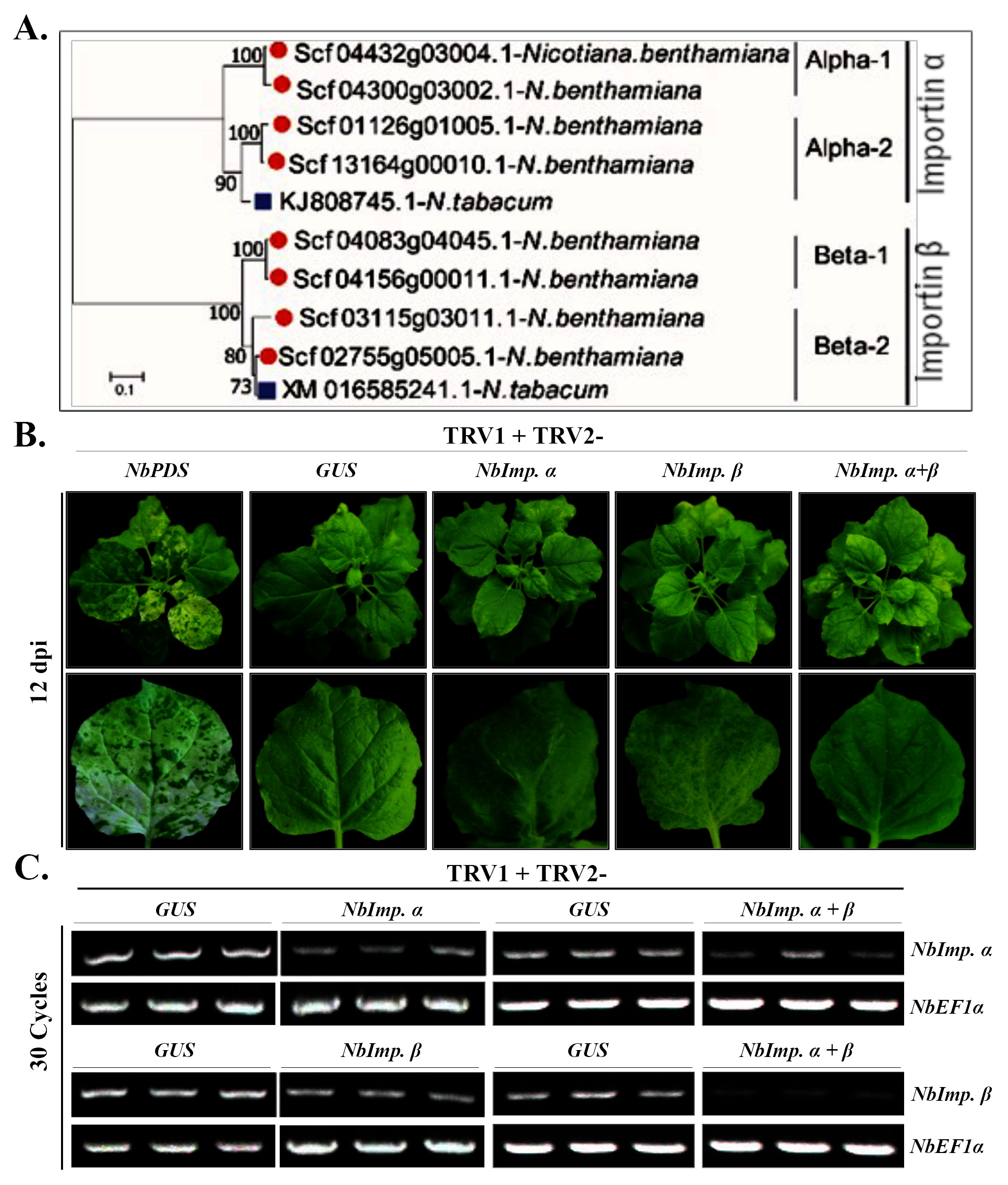

Supplement: S3 Fig — (A). Phylogenetic analysis all the encoding genes of importin α and importin β inN. benthamiana. Bar scale, 0.1. (B). Phenotype of silencing of NbImp. α, NbImp. β, and NbImp. α + β in N. benthamiana at 12 dpi. (C). The silencing efficiency were determined by the simi-quantitative PCR. The TRV-gus-inoculated plants were served as experimental control. All these PCRs were set to 30 cycles. (JPG) [file ppat.1011738.s003.jpg]

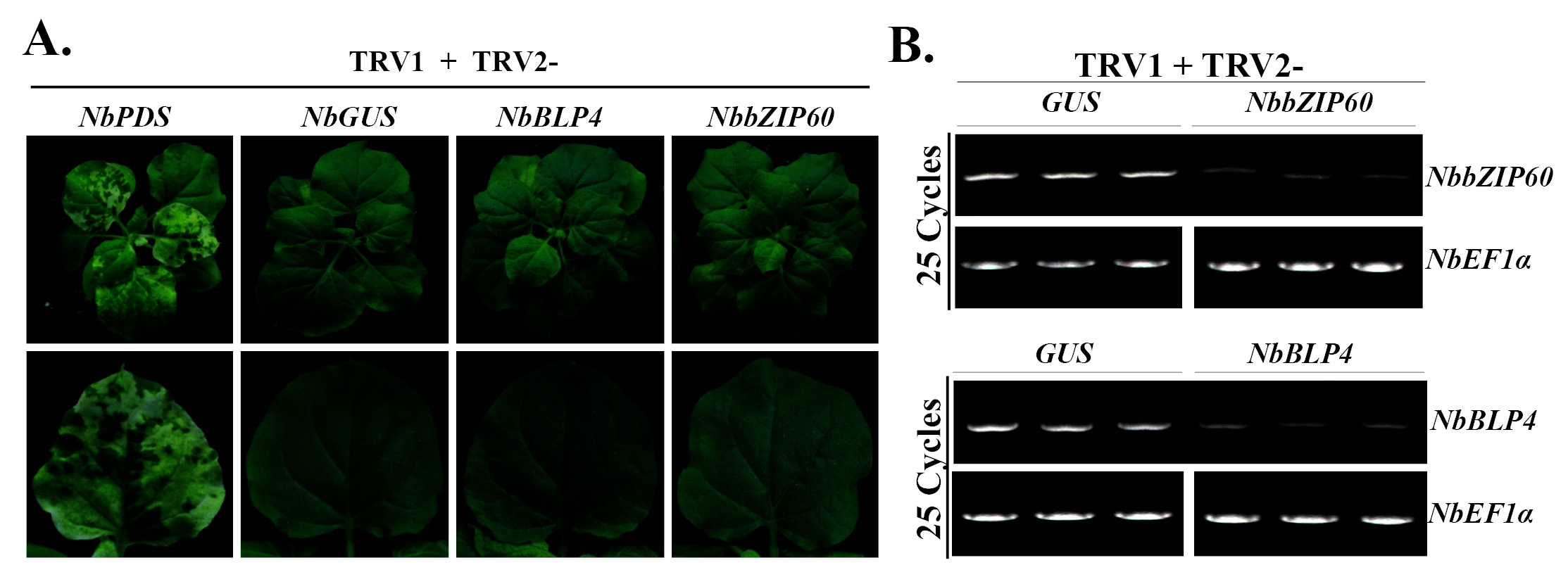

Supplement: S4 Fig — (A). Phenotype of silencing of NbPDS, NbGUS, NbBLP4, and NbbZIP60 at 12 dpi. (B). The silencing efficiencies were determined by the simi-quantitative PCR. All PCRs runs set to 25 cycles. (JPG) [file ppat.1011738.s004.jpg]

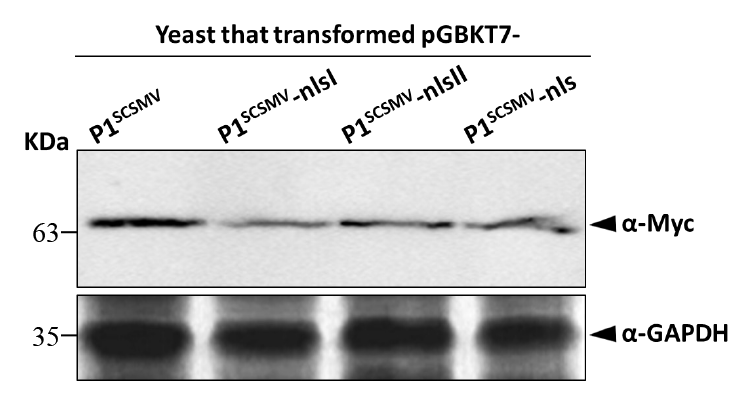

Supplement: S5 Fig — The glyceraldehyde-3-phosphate dehydrogenase (GAPDH) was treated as loading control. (TIF) [file ppat.1011738.s005.tif]

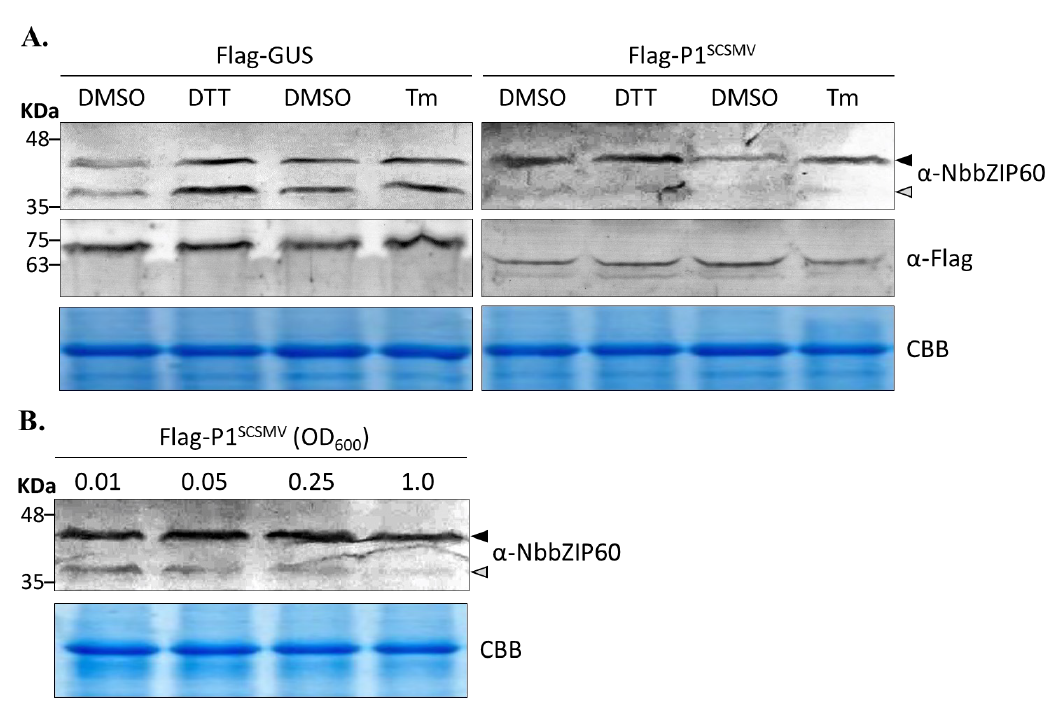

Supplement: S6 Fig — (A). P1SCSMV inhibited bZIP60U processing to a great extent in the presence and absence of the UPR inducers (DTT and tunicamycin). The concentration of DTT, tunicamycin (Tm), and DMSO were 2 mM, 5 μg/mL, and 0.1%. DMSO was used as a solvent to dissolve DTT and tunicamycin. After two days (48 h) of infiltration, and DTT, Tm, and DMSO were spayed to the target leaves. Two-hour (2 h) later, the leaves were harvested and its total proteins were extracted. The OD600 of Agrobacterium expressing Flag-GUS and Flag-P1SCSMV was set as 1.0. Polyclonal anti-NbbZIP60 and anti-FLAG antibodies were used to analyses proteins’ expression. (B). Gradient proteolytic processing of bZIP60U increased levels of P1SCSMV. Agrobacterium expressing Flag-P1SCSMV with different OD600 values infiltrated the N. benthamiana leaves. The internal spliced and un-spliced forms of bZIP60 were qualified using self-prepared anti-NbbZIP60 antibodies. (TIF) [file ppat.1011738.s006.tif]

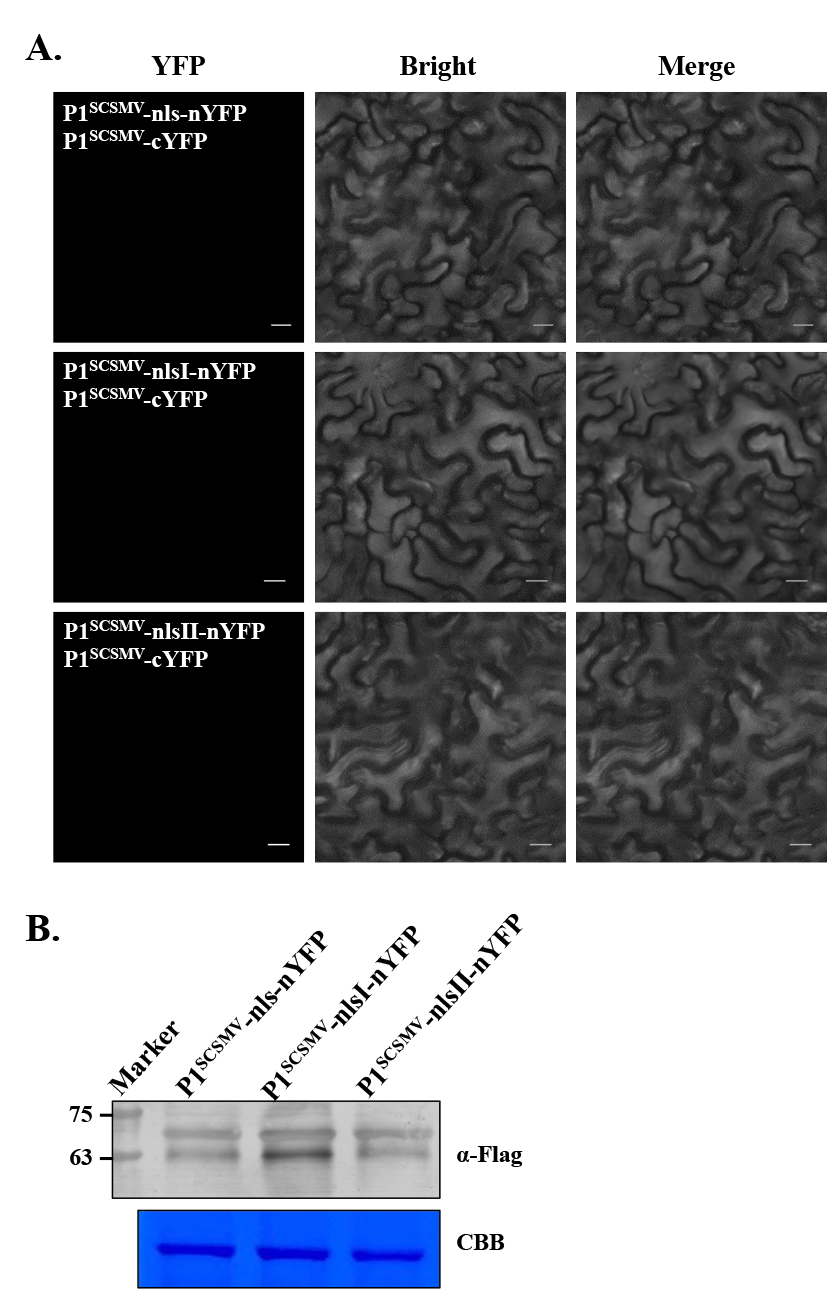

Supplement: S7 Fig — Bar scale, 20 μm. (TIF) [file ppat.1011738.s007.tif]

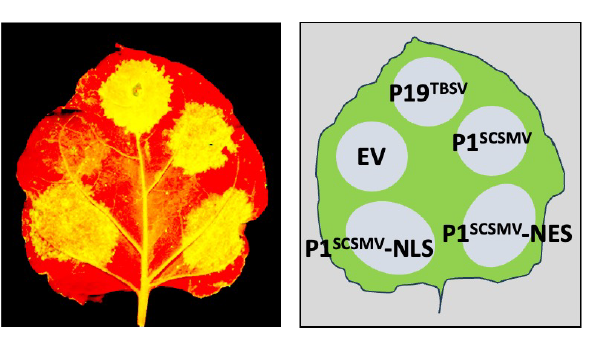

Supplement: S8 Fig — (TIF) [file ppat.1011738.s008.tif]
