# Supplementary material for: Sugarcane streak mosaic virus P1 protein inhibits unfolded protein response through direct suppression of bZIP60U splicing
Source: PLoS Pathog. 2023 Oct 26;19(10):e1011738. doi: 10.1371/journal.ppat.1011738 (PMC10697598; doi:10.1371/journal.ppat.1011738)
Supplement: S2 Table — (DOCX) [file ppat.1011738.s010.docx]

**Supplementary Table S2 List the sequences of the RNA probes used in this study.**

| **Gene fragments** | **Probe Name** | **Primer sequences (5’→3’)^a^** | **Primer length (bp)** |
| --- | --- | --- | --- |
| NbbZIP60U Fragment | *NbbZIP60U probe* | GGCTCCGAGGTTGATGATGACGACAAAGACAAGGAGAAGGGTTCCCAGTCGCCGACTGAGTCTAAGGACGGCTCCGACGAACTAAACAGTAACGATCCCGTCGATAAAAAGCGCAAGAGGCAATTGAGAAACAGGGATGCAGCTGTCAGGTCACGAGAGCGGAAGAAGTTGTATGTTAGGGATCTTGAGTTGAAGAGTAGATACTTTGAATCAGAGTGCAAGAGGTTGGGAATGGAAGTGCTAATGGTGCTTGTATGACCAAGCAGGAGTCTGCTGTGCTCTTGTTGGAATCCCTGCTGTTGGGTTCCCTGCTTTGGTTCTTGGGCATCATATGCCTGCTCATTCTTCCCAGCCAACCCTGGTTAATTCCAGAAGAAAATCAACGAAGCAGAAACCACCGTCTTCTGGTTCCAATAAAGGGAGGAAATAAGAATGGTCGGATTTTTGAGTTCGTGTCCTTCATGATGGGCAAGAGATGCAAAGCTTCAAGATCGAGGATGAAGTTCAATCCCCATTCTTTGGGAATTGTGAT | 532 |
| NbbZIP60U Mutant Fragment | *NbbZIP60U-M probe* | GGCTCCGAGGTTGATGATGACGACAAAGACAAGGAGAAGGGTTCCCAGTCGCCGACTGAGTCTAAGGACGGCTCCGACGAACTAAACAGTAACGATCCCGTCGATAAAAAGCGCAAGAGGCAATTGAGAAACAGGGATGCAGCTGTCAGGTCACGAGAGCGGAAGAAGTTGTATGTTAGGGATCTTGAGTTGAAGAGTAGATACTTTGAATCAGAGTGCAAGAGGTTGGGAATGGAAGTGCTAATGGTGCTTGTATGACCAA**CGUCCUCA**CTGCTGTGCTCTTGTT**CCUUAGG**CTGCTGTTGGGTTCCCTGCTTTGGTTCTTGGGCATCATATGCCTGCTCATTCTTCCCAGCCAACCCTGGTTAATTCCAGAAGAAAATCAACGAAGCAGAAACCACCGTCTTCTGGTTCCAATAAAGGGAGGAAATAAGAATGGTCGGATTTTTGAGTTCGTGTCCTTCATGATGGGCAAGAGATGCAAAGCTTCAAGATCGAGGATGAAGTTCAATCCCCATTCTTTGGGAATTGTGAT | 532 |
| CP^PVX^ | *CP^PVX^ probe* | ATGTCAGCACCAGCTAGCACAACACAGGCCACAGGGTCAACTACCTCAACTACCACAAAAACTGCAGGCGCAACTCCTGCCACAGCTTCAGGACTGTTCACCATCCCGGATGGGGATTTCTTTAGTACAGCCCGTGCTGTAGTAGCCAGCGATGCCGTTGCGACGAATGAGGACCTCAGCGAGATTGAGGCTGTCTGGAAGGACATGAAGGTGCCCACAGACACTATGGCACAGGCTGCTTGGGACTTAGTCAGACACTGTGCTGATGTGGGCTCATCTGCTCAAACAGAAATGATAGATACGGGTCCCTACTCCAACGGCATCAGCAGAGCCAGACTGGCAGCAGCAATCAAAGAGGTGTGCACACTTAGGCAATTTTGCATGAAGTATGCCCCAGTGGTATGGAACTGGATGCTGACTAACAACAGTCCACCTGCTAACTGGCAAGCGCAAGGTTTCAAGCCTGAGCACAAATTCGCTGCATTCGACTTCTTCAATGGAGTCACCAACCCAGCTGCCATCATGCCCAAAGAGGGGCTCATTCGGCCACCGTCTGAAGCTGAAATGAATGCTGCCCAAACTGCTGCCTTTGTGAAGATTACAAAGGCCAGGGCACAATCCAACGACTTTGCCAGCCTAGATGCAGCTGTCACTCGAGGTCGTATCACTGGAACAACAACCGCTGAGGCTGTTGTCACTCTACCACCACCATAA | 714 |

^a^ refers to the nucleotide sequence of the primers; The yellow background letters indicate the predicted two stem-loop regions in the *NbbZIP60U probe* and *NbbZIP60U-M probe*. The two panels of the red letters distributed in the *NbbZIP60U probe* showed the first base-paired stem-loop region, and the two panel of the green letters represents the second base-paired stem-loop region. The blue letters distributed in the *NbbZIP60U-M probe* showed the mutations.
