# Supplementary material for: Sugarcane streak mosaic virus P1 protein inhibits unfolded protein response through direct suppression of bZIP60U splicing
Source: PLoS Pathog. 2023 Oct 26;19(10):e1011738. doi: 10.1371/journal.ppat.1011738 (PMC10697598; doi:10.1371/journal.ppat.1011738)
Supplement: S3 Table — (PDF) [file ppat.1011738.s011.pdf]

Supplementary Table S3 Sequences feature of the *NbbZIP60U* mRNA

>Niben101Scf24096g00018.1 sequence match in blast db N.benthamiana Genome v1.0.1 predicted cDNA

TTTCCTTGCTATTTCCTCGGTTACCTTCTCTTTGGAGTTTGGACCCTTAACCTCTAAGAAATACGGCGTGTATCAC

GTGACTATTTATTGGCCCCATCCACAGGTCCCAAAATCGTAGCTGTAGCTGTAGTTCCCCCACCCCAAACCCCGTA

TTTCTTGTTGAATTTTGGTTCAATCATCCCAATCTGGGTTGTAGAATAGGCGATGGTGGATGACATCGATGATATC

GTTGGACACATCAATTGGGACGATGTAGATGACCTCTTCCACAACATTCTAGAGGATCCCGCCGACAATCTCTTCT

CTGCTCATGATCCGTCCGCGCCGTCTATCCAGGAGATCGAGCAGCTTCTCATGAACGATGATGAAATCGTCGGTCA

CGTGGCTGTCGGAGAGCCTGATTTTCAACTTGCTGACGACTTTCTCTCCGACGTGCTAGCCGATTCTCCTGTTCAG

TCCGATCTTTCTCACTCTGATAAAGTCATTGGATTCCCCGATTCCAAGGTTTCAAGTTGCTCAGAGGTTGATGATG

ACGACAAAGACAAGGAGAAGGTTTCCCAGTCGCGGATTGACTCTAAGGACGGCTCTGACGAACTAAACTGTGATGA

TCCCGTCGATAAAAAGCGTAAGAGGCAATTGAGAAACAGAGATGCAGCTGTCAGGTCACGAGAGCGGAAGAAGTTG

TATGTTAGGGATCTTGAGTTGAAGAGTAGATACTTTGAATCAGAGTGCAAGAGGTTGGGGTTAGTTCTCCAGTGCT

GTCTTGCAGAAAATCAAGCTTTGCGCTTCTCTTTGCAGAATGGCAATGCTAATGGTGCTTGTATGACCAAGCAGGA

GTCTG**CTGTGCTCTTGTTGGAATCCCTG**CTGTTGGGTTCCCTGCTTTGGTTCCTGGGCATCATATGCCTGCTCATT

CTTCCCAGCCAACCCTGGTTAATTCCAGAAGAAAATCAACGAAGCAGAAACCACGGTCTTCTGGTTCCGATAAAGG

GAGGAAATAAGGCTGGTCGGATTTTTGAGTTCCTGTCCTTCATGATGGGCAAGAGATGCAAAGCTTCAAGATCGAG

GATGAAGTTCAATCCCCATTCTTTGGGAATTGTTATGTGACTCATTGTGATCAAATCCTTTCCCTTGGTGGATCCT

GCTTCCTTTGAGTTCTTTAATTTGTATCATAGGCTTTCTGTATGAGTCTTTGCTTTTACATCTGAAGGACGCCGTT

CAGTGTTTTAGTTAATGACGTATGAGCTTACTAAGATCTATGCAACTTTTTAACAGTTCCAGCCTAAATTTGTTTC

GTCTCTGTTTTGCATTTTCTTTTCTTCATTCTTTCTGCCCGAGTGGTTGTTGTGGCATGGTAGCAGGATTACAAGG

CCACCACAAGATGCTACAAAATTTTCAAAATTATGAAATCGATGTGAAGATGAATGGTTAGATTTCTATTAAGTTT

GATATATAACGATTTAGCATCTTCAAGTATGTTCTCAGTTTGATGGTGAATTGAGGCTGTATGTGTTTTCCCTCTT

TTTTTCTACTCTATAATGGCTTGGAACGGAAAAATTGGATAGTCTGGAAGTGTATTGACAAATGAGAAAAGAAGCA

TCTCCGCAAATGTATGCACTCCTTTTCTTCTTTGGTATGGGTAAA

Note: Red letters indicate the intron sequences by splicing; Green box indicates the single-layer transmembrane domain; Underline marks the twin harpin stem-loop.

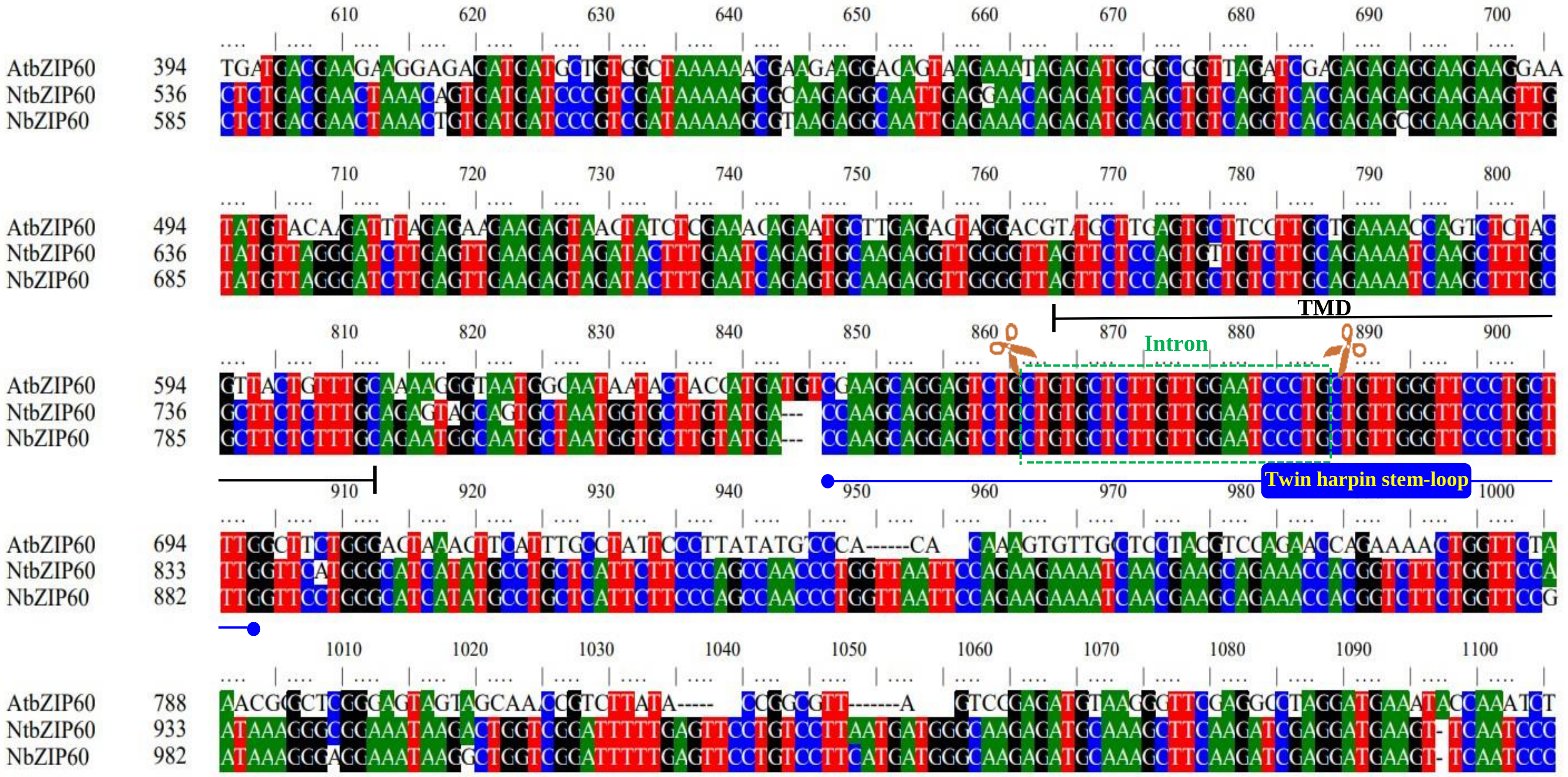
AtbZIP60
NtbZIP60
NbZIP60
TMD
Intron
Twin harpin stem-loop
